# Counteracting H3K4 methylation modulators Set1 and Jhd2 co-regulate chromatin dynamics and gene transcription

**DOI:** 10.1038/ncomms11949

**Published:** 2016-06-21

**Authors:** Saravanan Ramakrishnan, Srijana Pokhrel, Sowmiya Palani, Christian Pflueger, Timothy J. Parnell, Bradley R. Cairns, Srividya Bhaskara, Mahesh B. Chandrasekharan

**Affiliations:** 1 Department of Radiation Oncology, University of Utah School of Medicine, Salt Lake City, Utah 84112, USA; 2 Huntsman Cancer Institute, University of Utah School of Medicine, Salt Lake City, Utah 84112, USA; 3 Department of Oncological Sciences, University of Utah School of Medicine, Salt Lake City, Utah 84112, USA

## Abstract

Histone H3K4 methylation is connected to gene transcription from yeast to humans, but its mechanistic roles in transcription and chromatin dynamics remain poorly understood. We investigated the functions for Set1 and Jhd2, the sole H3K4 methyltransferase and H3K4 demethylase, respectively, in *S. cerevisiae*. Here, we show that Set1 and Jhd2 predominantly co-regulate genome-wide transcription. We find combined activities of Set1 and Jhd2 via H3K4 methylation contribute to positive or negative transcriptional regulation. Providing mechanistic insights, our data reveal that Set1 and Jhd2 together control nucleosomal turnover and occupancy during transcriptional co-regulation. Moreover, we find a genome-wide co-regulation of chromatin structure by Set1 and Jhd2 at different groups of transcriptionally active or inactive genes and at different regions within yeast genes. Overall, our study puts forth a model wherein combined actions of Set1 and Jhd2 via modulating H3K4 methylation−demethylation together control chromatin dynamics during various facets of transcriptional regulation.

## Abstract (web summary)

Histone H3K4 methylation affects gene transcription from yeast to humans. Here, the authors show that Set1 and Jhd2, the only H3K4 methyltransferase and H3K4 demethylase, respectively, in budding yeast, co-regulate both positive and negative transcription mediated by nucleosomal turnover and occupancy.

## Graphical abstract

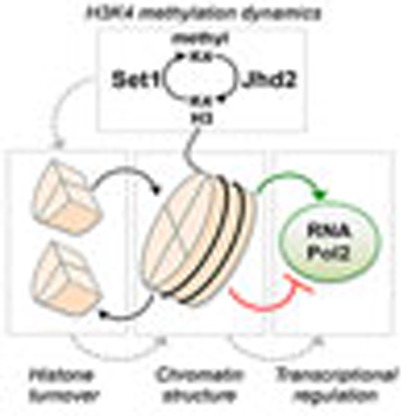

Histone post-translational modifications regulate chromatin structure and dynamics during many nuclear processes. One modification—histone H3 lysine-4 methylation (H3K4me)—is well-studied in the context of gene transcription. H3K4me exists in three states—monomethyl (me1), dimethyl (me2) or trimethyl (me3). H3K4me1 is connected to transcriptional enhancer functions and to gene repression in metazoans^1^,^2^, and linked to nucleosome dynamics and chromatin remodeling at stress-responsive genes in yeast^3^. H3K4me2 is linked to ongoing transcription as well as gene repression in yeast^4^,^5^,^6^,^7^. In general, H3K4me3 is considered a universal hallmark of active transcription, as genome-wide studies from yeast to humans have shown a strong correlation between active transcription and the occurrence of H3K4me3 around transcriptional start sites (TSSs)^8^,^9^,^10^. Specific protein domains or ‘reader' modules present in many chromatin modifying or remodeling complexes, which function to alter the local chromatin structure, recognize the H3K4 methyl marks^11^. Nevertheless, functions for H3K4 methylation in regulating transcription and chromatin structure are far from completely understood.

Studies in *Saccharomyces cerevisiae* or budding yeast have provided valuable insights into the regulation and functions of H3K4 methylation. Set1 is the sole H3K4 methyltransferase in yeast^12^,^13^, where it is present along with other regulatory proteins in a complex termed COMPASS (complex associated with Set1)^14^,^15^. Set1 is targeted to the sites of transcription initiation by virtue of its association with the serine-5 phosphorylated form of RNA polymerase II (Pol2)^16^. Set1 and the H3K4 residue are required for gene expression at many yeast loci and under various growth regimes^7^,^17^,^18^,^19^,^20^,^21^. Jhd2 is the JARID-family H3K4 demethylase in yeast^22^,^23^,^24^,^25^. Jhd2 also functions at transcribed genes^26^ and has a key role in activating transcription at protein-coding genes during sporulation^27^. Therefore, Set1, Jhd2 and H3K4 methylation have a positive role during transcription.

Set1 and its associated COMPASS subunits are required to repress transcription at protein-coding and non-coding RNA genes under normal and stress conditions^5^,^21^,^28^,^29^. Recent studies have suggested a significant role for Set1-mediated H3K4 methylation during gene repression in yeast^21^. Jhd2 is essential for repressing antisense and intergenic non-coding RNA transcription during gametogenesis^27^. Thus, Set1, Jhd2 and H3K4 methylation have all been also linked to transcriptional repression. In these reported studies, Set1 or Jhd2 is proposed to regulate transcription, either via the control of antisense transcription or by influencing nucleosome organization, or both^5^,^21^. Collectively, studies described above have ascribed either a positive or a negative role for Set1 or Jhd2 during transcriptional regulation at certain yeast genes, but common functional target genes of Set1 and Jhd2 are not known. Also, whether Set1 and Jhd2 via modulating H3K4 methylation regulate chromatin structure and dynamics remains to be understood.

Using the genetic model system of *S. cerevisiae*, we examined the role for its single H3K4 methyltransferase, Set1, and its sole H3K4 demethylase, Jhd2, on gene transcription and chromatin structure through targeted and genomic studies. Contrary to a simplistic model, we find that counteracting enzymes Set1 and Jhd2 mostly co-regulate transcription under normal growth conditions. We also report that the enzymatic activities of Set1 and Jhd2 through modulating H3K4 methylation contribute to positive or negative co-regulation of transcription at shared target genes. Importantly, Set1 and Jhd2 work together to impact nucleosomal turnover and occupancy at their common target genes. We report that Set1 and Jhd2 also co-regulate nucleosomal turnover and chromatin structure genome-wide across numerous genes in yeast. Overall, this study uncovers a novel mechanism wherein the joint activities of Set1 and Jhd2, via H3K4 methylation and demethylation, modulate nucleosomal turnover, chromatin dynamics and RNA Pol2 functions during transcriptional regulation.

## Results

### Set1 and Jhd2 co-regulate transcription at target genes

**Figure 1:**
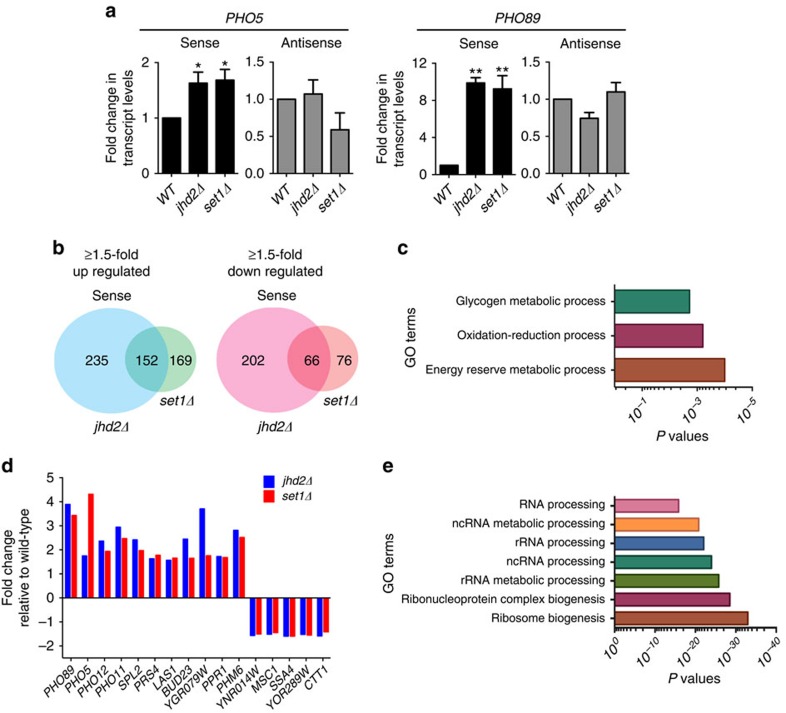
Counteracting enzymes Set1 and Jhd2 function to positively or negatively co-regulate transcription in yeast. (**a**) Strand-specific qPCR measurement of *PHO5* or *PHO89* sense and antisense transcription. Fold-change in transcript levels in a mutant is shown relative to WT strain (WT) (set as 1). Error bars denote±s.e.m. from three independent experiments (*n*=3). Statistical significance calculated using the Student's *t*-test, *P-value ≤0.02, **P-value <0.001. (**b**) Venn diagrams showing the number of up- or down regulated sense transcripts unique to and common to *jhd2Δ* and *set1Δ* mutants. Intersection *P*-value <10^−4^ (hypergeometric test). (**c**) GO terms enriched in sense transcripts down regulated in both *set1Δ* and *jhd2Δ* mutants. (**d**) Set1 and Jhd2 either positively or negatively regulate transcription at the phosphate-responsive genes. Fold-change in total RNA-seq reads obtained in *jhd2Δ* or *set1Δ* mutant relative to WT for various phosphate-responsive genes are shown. (**e**) GO terms enriched in sense transcripts up regulated in both *set1Δ* and *jhd2Δ* mutants.

De-repression of *PHO5*, a phosphate-responsive gene, occurs in strain lacking either Set1 H3K4 methyltransferase (*set1Δ*)^30^ or Jhd2 H3K4 demethylase (*jhd2Δ*)^26^. Non-coding antisense transcription initiating from 3′ regions occurs at *PHO5* and other phosphate-responsive genes^31^,^32^. We asked whether opposing enzymes Set1 and Jhd2 work in a contrasting fashion to control either sense or antisense *PHO5* transcription. Strand-specific quantitative PCR (qPCR) showed that only *PHO5* sense transcript levels were increased in *set1Δ* and *jhd2Δ* compared to the control (Fig. 1a). Similar results were obtained for *PHO89*, another phosphate-responsive gene (Fig. 1a). Therefore, these results revealed an unexpected finding that both Set1 and Jhd2 are negative regulators that repress *PHO5* and *PHO89* sense transcription.

We then examined Set1 and Jhd2 functions in a global scale using an RNA sequencing (RNA-seq) experiment, where we measured stranded expression in wild type (WT), *set1Δ* and *jhd2Δ* strains. Bioinformatics analysis showed a change in the steady state levels of many sense and antisense transcripts in *set1Δ* and *jhd2Δ* compared to the control (that is, ≥1.5-fold up or down-regulation at a false discovery rate (FDR) ≤5%) (Table 1; Supplementary Data 1 and 2). We then identified transcripts differentially expressed in *set1Δ* and *jhd2Δ*. At a threshold of ≥1.5-fold change (FDR ≤5%), a very small number of transcripts showed differential expression in *set1Δ* compared to *jhd2Δ* (Table 2; Supplementary Fig. 1). In a striking contrast, expression of a common set of 152 sense and 14 antisense transcripts were up regulated and another set of 66 sense and 18 antisense transcripts were down regulated in both *set1Δ* and *jhd2Δ* (Fig. 1b; Supplementary Fig. 2). Thus, these gene expression studies while identifying distinct target genes of Set1 or Jhd2 also uncovered a large common group of target genes where both Set1 and Jhd2 function as either positive or negative regulators of transcription. Importantly, they showed for the first time that combined regulation of transcription by Set1 and Jhd2 is more pervasive than the anticipated counter-regulation.

To identify biological processes co-regulated by Set1 and Jhd2, we performed gene ontology (GO) analysis, which showed genes for glycogen or energy reserve metabolism were enriched in sense transcripts down regulated in *set1Δ* and *jhd2Δ* (*P*-value ≤0.001, hypergeometric distribution) (Fig. 1c; Supplementary Data 3). A closer inspection of the down regulated genes revealed *SER3* and *CHA1* genes involved in serine metabolism (Supplementary Fig. 3a), and well-studied models for intergenic transcription and chromatin-mediated regulation^33^, respectively. Down-regulation of *SER3* was not due to increased expression of *SRG1* (ref. 34) (Supplementary Fig. 3b), suggesting direct regulation of *SER3* transcription by Set1 and Jhd2. Thus, Set1 and Jhd2 positively regulate transcription at a shared set of target genes, such as, those involved in amino acid or glycogen metabolism.

Since Set1 and Jhd2 negatively regulate *PHO5* and *PHO89* sense transcription (Fig. 1a), we compared the up- or down regulated transcripts common to *set1Δ* and *jhd2Δ* to all 127 phosphate-responsive genes in yeast^33^,^35^,^36^. Sense transcript levels for genes involved in inorganic phosphate regulation or utilization and polyphosphate metabolism were increased in *set1Δ* and *jhd2Δ* (Fig. 1d). A decrease in sense transcript levels for certain phosphate-responsive genes was also observed in these mutants (for example, *MSC1*, *CTT1*) (Fig. 1d). Therefore, Set1 and Jhd2 promote as well as repress transcription at phosphate-responsive genes, when cells are in YPD medium containing abundant inorganic phosphate^37^. Set1 represses transcription at ribosome biogenesis or Ribi genes^21^. GO analysis of the shared sense transcripts up regulated in *set1Δ* and *jhd2Δ* showed enrichment for genes involved in ribosomal RNA processing and ribosome biogenesis (*P*-value ≤10^−20^, hypergeometric distribution) (Fig. 1e; Supplementary Data 3). Therefore, Set1 and Jhd2 are negative regulators of transcription at a sub-set of Ribi genes in addition to phosphate-responsive genes.

**Figure 2:**
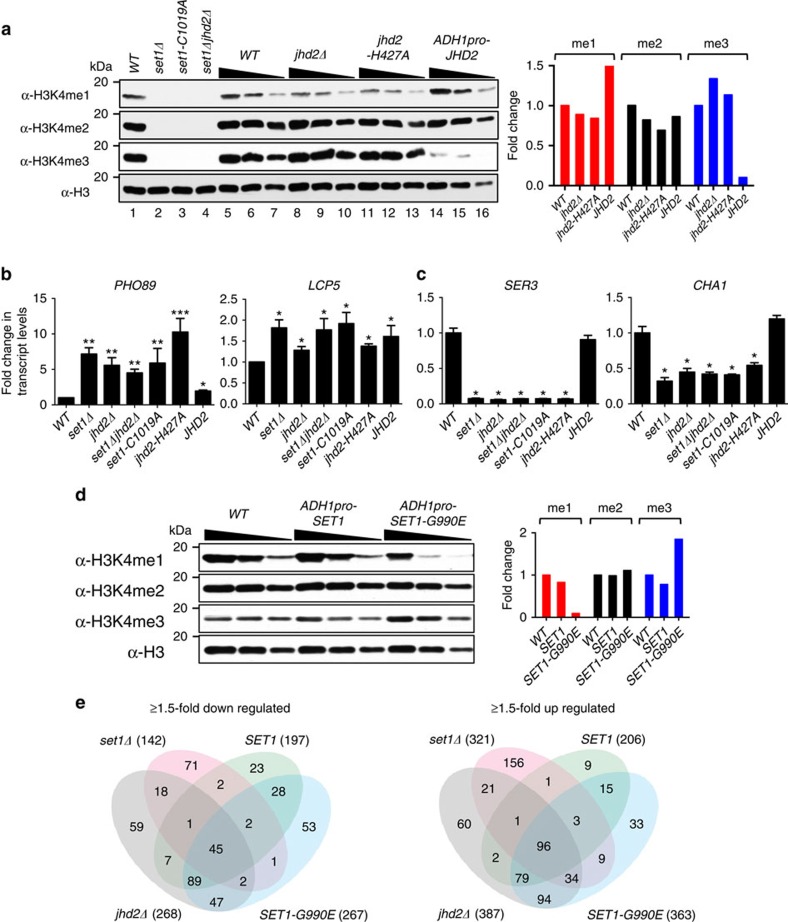
Transcriptional co-regulation is dependent on Set1 and Jhd2 catalytic activities and H3K4 methylation. (**a**) Western blots for H3K4me1, H3K4me2 and H3K4me3 in WT, deletion mutants (*set1Δ*, *jhd2Δ* and *set1Δ jhd2Δ*), catalytic-dead mutants (*set1-C1019A* and *jhd2-H427A*) and Jhd2 overexpressing strain (*ADH1pro-JHD2*) are shown. Histone H3 serves as loading control. Twofold serial dilutions of cell extracts from the indicated strains were applied in lanes 5–16. Graph shows quantitation of western blots using densitometry. Fold-change in a H3K4 methyl mark level normalized to the total H3 level in a mutant is shown relative to that in control WT (set as 1). (**b**) Fold-change in *PHO89* and *LCP5* sense transcript levels in the indicated mutants relative to WT are shown. For (**b**,**c**), error bars denote ±s.e.m. from four independent experiments (*n*=4). Statistical significance calculated using the Student's *t*-test. *PHO89*: *P-value <0.05, **P-value <0.005, ***P-value <10^−5^; *LCP5*: *P-value ≤0.005. (**c**) Fold-change in *SER3* and *CHA1* transcript levels in the indicated mutants relative to WT are shown. *SER3*: *P-value <10^−5^, *CHA1*: *P-value <10^−3^. (**d**) Western blots for bulk H3K4 methylation and H3 levels in WT strain or strains overexpressing SET1 or hyperactive SET1-G990E. Graph shows quantitation of western blots using densitometry. (**e**) Four-way Venn diagrams showing the number of shared up- or down-regulated sense transcripts in each combination of indicated deletion or overexpression mutant strains. The total number of up- or down-regulated transcripts in each mutant is indicated in parentheses. For (**a**–**d**), see Supplementary Figs 20 and 21 for full-length image of blots.

Since the effects on gene expression for *set1Δ* and *jhd2Δ* were largely identical, we asked whether a combined double mutant would produce a synergistic effect. We created a yeast strain deleted for both *SET1* and *JHD2* (*set1Δjhd2Δ*) (Fig. 2a, lane 4). qPCR showed an increase in the transcript levels for the co-repressed target genes (*PHO5*, *PHO89*, and Ribi genes *LCP5* and *DBP8*) and a decrease for the co-activated target genes (*SER3* and *CHA1*) in *set1Δjhd2Δ* double mutant compared to the WT control (Fig. 2b,c; Supplementary Fig. 4). However, no additive effect on target gene expression or repression was observed in the *set1Δjhd2Δ* double mutant when compared to the single mutants (*set1Δ* or *jhd2Δ*) (Fig. 2b,c; Supplementary Fig. 4). Therefore, Set1 and Jhd2 contribute equally to positive or negative transcriptional co-regulation at their shared target genes.

### Transcription is dependent on Set1 and Jhd2 activities

We asked whether enzymatic activity of Set1 or Jhd2 or their common target (H3K4) is required for target gene regulation. We generated strains expressing catalytic-dead alleles of *SET1* or *JHD2*, *set1-C1019A*^38^ or *jhd2-H427A*^22^, respectively (Fig. 2a, lanes 3, 11–13). We also generated strains expressing WT H3 or an H3 mutant allele with a lysine to arginine substitution at position 4 (H3K4R). qPCR showed increased expression of the co-repressed target genes and decreased expression of the co-activated target genes in *set1-C1019A* and *jhd2-H427A* mutants compared to the control—a result very similar to that obtained for the *set1Δ* and/or *jhd2Δ* deletion mutants (Fig. 2b,c; Supplementary Fig. 4). Similar results were obtained for the *H3K4R* mutant (Supplementary Fig. 5). Collectively, these findings mechanistically link the catalytic activities of Set1 and Jhd2, and the H3K4 residue to transcriptional co-regulation at the shared target genes.

To examine the role for H3K4 methylation in target gene regulation, we overexpressed the Jhd2 demethylase in yeast, which decreased global H3K4me3 and subtly increased bulk H3K4me1 (Fig. 2a, lanes 14–16). qPCR showed that transcripts for co-repressed target genes were generally increased upon Jhd2 overexpression (Fig. 2b; Supplementary Fig. 4), suggesting a role for H3K4me1 and H3K4me3 in their transcriptional regulation. However, unlike in the *set1Δ* mutant, decrease in bulk H3K4me3 upon Jhd2 overexpression did not decrease *SER3* and *CHA1* transcript levels (Fig. 2c). Therefore, Set1 and Jhd2 co-activate target gene expression either through a general maintenance of H3K4 methylation or alternatively, via maintaining H3K4me1 and/or H3K4me2 levels.

To further explore the role for H3K4 methylation in target gene regulation, we generated yeast strains constitutively expressing Set1-G990E, a dominant hyperactive form of Set1 (ref. 38), or expressing WT Set1. Expression of Set1-G990E increased global H3K4me3 at the expense of bulk H3K4me1 (Fig. 2d). We examined genome-wide gene expression by stranded RNA-seq in Set1 or Set1-G990E overexpression strains, which showed altered steady state levels of sense and antisense transcripts (Supplementary Fig. 6; Supplementary Data 1 and 2). Comparing transcripts altered in Set1 or Set1-G990E overexpression strain to those altered in *set1Δ* or *jhd2Δ* revealed a large set of 96 up regulated and 45 down regulated sense transcripts common to all four strains (Fig. 2e), and very few shared antisense transcripts (Supplementary Fig. 6b). GO analysis showed Ribi genes (*P*-value <10^−20^, hypergeometric distribution) and energy reserve metabolism and glycogen biosynthesis genes (*P*-value <10^−4^, hypergeometric distribution) enriched in sense transcripts up- or down regulated in all four strains, respectively (Supplementary Fig. 7a,b; Supplementary Data 3). Therefore, transcriptional control at certain shared Set1 and Jhd2 target genes is very sensitive to any loss or gain in H3K4 methylation.

### Set1 and Jhd2 co-localize and regulate H3K4 methylation

We then examined the global distribution and levels of H3K4me1, H3K4me2 and H3K4me3 at shared target genes in *set1Δ* and *jhd2Δ* mutants. We used a modified ChIP-seq (chromatin immunoprecipitation and DNA sequencing) procedure^39^,^40^, where defined quantities of a reference epigenome (*Candida glabrata*) were mixed with the samples before immunoprecipitation, which allowed us to accurately calibrate and quantitatively measure the enrichment of H3K4 methyl marks in WT and *jhd2Δ* strains. Before using *C. glabrata* as the reference epigenome, we confirmed the occurrence of H3K4 methylation in this organism (Supplementary Fig. 8).

**Figure 3:**
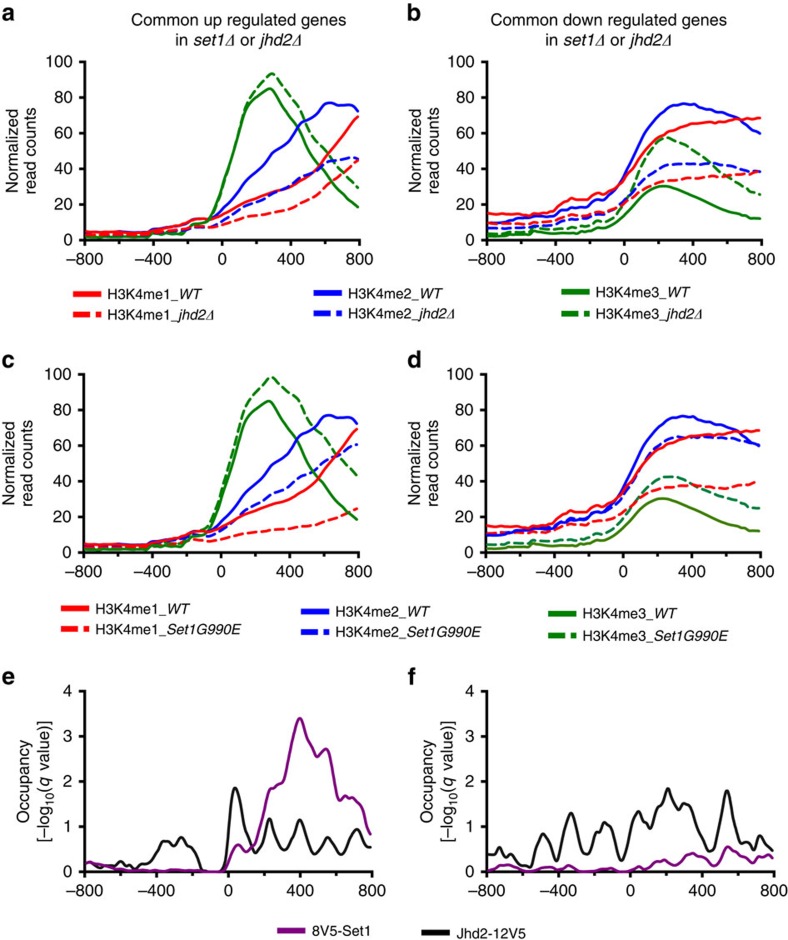
Set1 and Jhd2 localize to their shared target genes and regulate H3K4 methylation. (**a**,**b**) Normalized-read counts profiles for H3K4 monomethyl (me1), dimethyl (me2) and trimethyl (me3) marks across 800 bp region upstream (−) or downstream (+) of the TSSs (0) at target genes co-repressed (**a**) or co-activated (**b**) by Set1 and Jhd2 in WT and *jhd2Δ* null mutant. (**c**,**d**) Normalized-read counts profiles for H3K4me1, H3K4me2 and H4K4me3 marks across regions, as described for (**a**,**b**), at genes co-repressed (**c**) or co-activated (**d**) by Set1 and Jhd2 in WT and *Set1-G990E* gain-of-function or hyperactive mutant. (**e**,**f**) Statistical enrichment (qpois) profiles for 8V5-tagged Set1 or 12V5-tagged Jhd2 occupancies across 800 bp region upstream (−) or downstream (+) of the TSSs (0) at the Set1 and Jhd2 co-repressed (**e**) or co-activated (**f**) target genes.

Analysis of quantitative ChIP-seq (qChIP-seq) data showed that genes co-repressed by Set1 and Jhd2 were enriched with H3K4me3 at their 5′-ends and H3K4me1 and H3K4me2 at their 3′-ends in the WT strain (Fig. 3a), an occupancy profile considered typical for H3K4 methylation at active yeast genes^41^. The Set1 and Jhd2 co-activated target genes contained higher levels of H3K4me1 and H3K4me2 than H3K4me3 (Fig. 3b), revealing a novel class of active yeast genes containing an atypical H3K4 methylation occupancy profile significantly different from that of an average active yeast gene^41^ and at the co-repressed target genes (Fig. 3a). At both co-repressed and co-activated target genes, H3K4me3 was increased and H3K4me1 and H3K4me2 were decreased in *jhd2Δ* compared to the control (Fig. 3a,b; Supplementary Fig. 9a,b). This finding suggests that Jhd2 demethylates H3K4me3 to maintain relative amounts of the three H3K4 methyl marks at shared target genes. Next, analyses of published ChIP-seq datasets for H3K4 methylation in *set1Δ* mutant^42^ showed no enrichment across all shared target genes (Supplementary Fig. 10a,b). In addition, qChIP-seq of H3K4 methylation in the strain expressing hyperactive Set1-G990E showed increased H3K4me3, and decreased H3K4me1 and H3K4me2 at co-repressed or co-activated target genes (Fig. 3c,d; Supplementary Fig. 9a,b). These results confirm that Set1 regulates H3K4 methylation at shared target genes. Interestingly, H3K4me1 and H3K4me2 are reduced at target genes in all three mutants (*set1Δ*, *jhd2Δ* and Set1-G990E), which implicate a functional role for H3K4me1 and/or H3K4me2 marks in Set1 and Jhd2-mediated transcriptional co-regulation. Taken together, these results demonstrate that Set1 and Jhd2 control H3K4 methylation dynamics during transcriptional co-regulation at their shared target genes.

We performed ChIP-seq to then map the genome-wide binding locations for Set1 and Jhd2 on chromatin. To increase ChIP efficiency of these proteins, chromatin was prepared from nuclei isolated from formaldehyde cross-linked yeast cells and digested with micrococcal nuclease (MNase). Analysis of ChIP-seq data showed physical association of both Set1 and Jhd2 at co-activated or co-repressed target genes (Fig. 3e,f). Moreover, Jhd2 occupancy was enriched at TSSs and promoter upstream regions and Set1 occupancy was enriched over coding regions of the co-repressed target genes (Fig. 3e). Set1 and Jhd2 occupancies were dispersed across the co-activated target genes, albeit Jhd2 levels were much higher than Set1 (Fig. 3f). Therefore, Set1 and Jhd2 directly associate with and modulate H3K4 methylation levels at target genes during transcriptional co-regulation.

### Set1 and Jhd2 co-regulate chromatin structure

**Figure 4:**
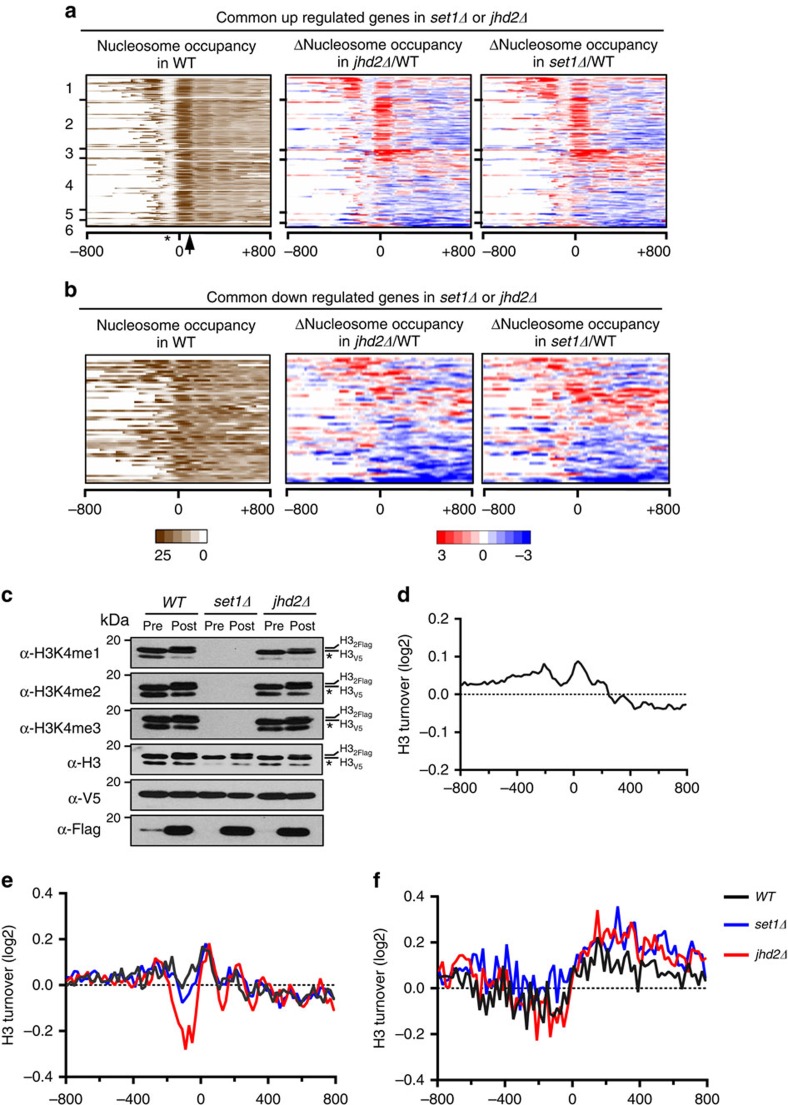
Set1 and Jhd2 co-regulate nucleosomal occupancy and histone turnover at their shared target genes. (**a**,**b**) Heat maps for nucleosome occupancy ratios (Δnucleosome occupancy) between *jhd2Δ* and *WT* or between *set1Δ* and *WT* at genes either co-repressed (**a**) or co-activated (**b**) by Set1 and Jhd2. Red, gain in nucleosome occupancy; blue, loss in nucleosome occupancy. Genes in rows are organized into six groups using *k*-means clustering. Columns represent 50 bp windows over ±800 bp regions relative to the TSS (0). Windows overlapping neighbouring genes were excluded. Total reads for mononucleosomal DNA in WT show the native state of nucleosome occupancy (leftmost panel). Asterisk, NDR; black arrow, the well-positioned +1 nucleosome. (**c**) Western blots showing RITE. *WT*, *set1Δ* and *jhd2Δ* strains expressing histone H3 tagged with V5-*LoxP*-HygMX-*LoxP*-2Flag cassette were grown overnight in YPD medium and then grown to saturation followed by addition of β-estradiol to induce tag switch. Saturated cultures were re-inoculated into fresh medium to release cells into the cell cycle in the presence of β-estradiol. Cells were arrested in G1 using α-factor. Cells before addition of β-estradiol (pre) and β-estradiol-treated G1 cells (post) were collected for whole-cell lysate preparation and detection of old (V5) and new (Flag) histone H3. The levels of H3K4me1, H3K4me2, H3K4me3 and total histone H3 in the indicated strains are shown. *2Flag*, two copies of Flag tag; asterisk, truncated H3. See Supplementary Fig. 22 for full-length image of blots. (**d**) The average mean profile for histone H3 turnover across 800 bp region upstream (−) or downstream (+) of the TSSs (0) at all yeast genes. (**e**,**f**) Mean profiles for histone H3 turnover across regions (as described for **d**) at genes co-repressed (**e**) or co-activated (**f**) by Set1 and Jhd2 in WT, *set1Δ* and *jhd2Δ* strains.

To uncover the underlying molecular mechanisms, we asked whether Set1 and Jhd2 control chromatin structure at their common target genes. To test this possibility, we explored the impact of loss of either Set1 or Jhd2 on nucleosome occupancy at a global scale. We examined nucleosomal architecture across the genome by high-throughput sequencing of mononucleosomes isolated from WT, *set1Δ* and *jhd2Δ* strains following MNase digestion (Supplementary Fig. 11). Nucleosome occupancy maps were generated from the MNase-seq reads obtained for each strain. We subtracted the normalized nucleosome occupancy in a mutant strain from that in the control strain to generate a differential map (Fig. 4a,b, panels labelled Δnucleosome occupancy). To examine promoters, we scored nucleosome occupancy in windows flanking the TSSs (Fig. 4a,b).

To aid in visualization, we sorted the nucleosomal occupancy changes occurring in *set1Δ* and *jhd2Δ* mutants relative to WT at the TSS across all yeast genes into six categories using a *k*-means clustering algorithm. The co-repressed target genes contain well-positioned nucleosomes, including a very well-positioned +1 nucleosome, and a distinct nucleosome-depleted region (NDR) upstream of TSS (Fig. 4a, panel labelled nucleosome occupancy in WT). This chromatin organization is considered typical for yeast genes and it is similar to that at ‘growth' or housekeeping genes^43^. In contrast, co-activated target genes lack a well-defined NDR and contain poorly aligned nucleosomes (Fig. 4b). This chromatin architecture resembles that reported for the ‘stress' genes^43^. These distinct chromatin conformations while reflecting the intrinsic nature of target genes also highlight the importance of Set1 and Jhd2 in co-regulating different types of gene ontologies represented by the respective target genes.

To determine the impact of Set1 and Jhd2 on nucleosomal or chromatin architecture at target genes, we examined the differential maps that showed loss or gain of nucleosome occupancy in the *set1Δ* and *jhd2Δ* mutants relative to the WT strain. At co-repressed target genes (Fig. 4a, panels labelled Δnucleosome occupancy), deletion of *SET1* or *JHD2* induced strikingly similar changes to nucleosomal architecture; a similar effect was also observed at co-activated target genes, where changes to the occupancies of nucleosomes were almost identical regardless of the loss of Set1 or Jhd2 (Fig. 4b). In general, both nucleosomal loss and gain were observed in *set1Δ* and *jhd2Δ*; however, effects were more consistent in co-repressed target genes, with prominent nucleosomal gains at −1 and/or +1 nucleosomes. The consistency of effects observed in the co-repressed target genes may simply reflect that these genes have a strong chromatin organization, as opposed to the lack of a consensus chromatin organization at the co-activated target genes.

Collectively, results from MNase-seq demonstrate that Set1 and Jhd2, despite their counteracting enzymatic activities, act in a concerted fashion to control nucleosome occupancy over regulatory and/or coding regions during transcriptional co-regulation.

### Set1 and Jhd2 co-regulate nucleosome turnover

Histone exchange (also known as histone or nucleosome turnover) refers to disassembly and reassembly of nucleosomes during DNA replication and transcription^44^. Histone turnover is most frequently associated with nucleosomes flanking gene promoters^45^ and is correlated with H3K4 methylation^46^. We asked whether Set1 and Jhd2 might control histone turnover to co-regulate nucleosomal occupancy and transcription at their target genes. Hence, we used recombination-induced tag exchange (RITE)^47^ method to differentially tag old and newly synthesized histone H3 within yeast cells. We tagged one of the two loci coding for histone H3 with a V5 epitope flanked by two *LoxP* recombination sites and followed by two Flag epitopes. In addition to the control WT strain, we created the RITE system in *set1Δ* and *jhd2Δ* mutants. To measure replication-independent and transcription-coupled nucleosome turnover, we arrested cells in G1 phase using α-factor. Epitope tag switch was accomplished by the addition of β-estradiol, which activated a constitutively expressed Cre recombinase fused to human oestrogen binding domain (Fig. 4c). To examine genome-wide nucleosome turnover, we isolated chromatin from G1-arrested cells for immunoprecipitation using anti-V5 or anti-Flag antibodies, which was followed by exonuclease digestion to trim DNA fragments before library preparation and sequencing (ChIP-nexus^48^). We calculated nucleosome turnover in control and mutant strains by determining the ratio between the Flag (new histone H3) and V5 (old histone H3) ChIP signal.

Analysis of ChIP-nexus data from the WT strain showed a high rate of nucleosome turnover around promoters with a progressive reduction over the coding regions of all yeast genes (Fig. 4d), agreeing with published results^45^ and validating our approach. We then examined nucleosome turnover at co-repressed or co-activated target genes in WT, *set1Δ* and *jhd2Δ* strains. At co-repressed target genes, a dramatic decrease in nucleosome turnover at promoters was observed in *jhd2Δ*, and to a lesser degree also in *set1Δ*, when compared to the control (Fig. 4e; Supplementary Fig. 12a). A low nucleosomal turnover rate could occur from poor histone H3 deposition or from rapid ejection of nucleosomes. Therefore, absence of Set1 or Jhd2 reduces histone turnover at promoters causing nucleosomal loss, which likely allows the binding of activators or RNA Pol2 and up-regulating transcription at co-repressed target genes. At co-activated genes, a higher rate of nucleosomal turnover was observed over coding regions, especially when compared to the average nucleosome turnover across all yeast genes and co-repressed target genes (compare Fig. 4d–f). A high nucleosomal turnover could result from increased histone H3 deposition or from increased cycles of nucleosome disassembly and reassembly. Deletion of *SET1* or *JHD2* increased nucleosomal turnover across the coding regions of co-activated target genes (Fig. 4f; Supplementary Fig. 12b). Therefore, loss of Set1 or Jhd2 further increases the already high nucleosomal turnover over coding regions, which likely impedes RNA Pol2 progression causing down-regulation of transcription at co-activated target genes.

Overall, results from RITE−ChIP-nexus experiments demonstrate that Set1 and Jhd2 co-regulate nucleosome turnover at their target genes.

### Set1 and Jhd2 co-regulate nucleosomal occupancy

**Figure 5:**
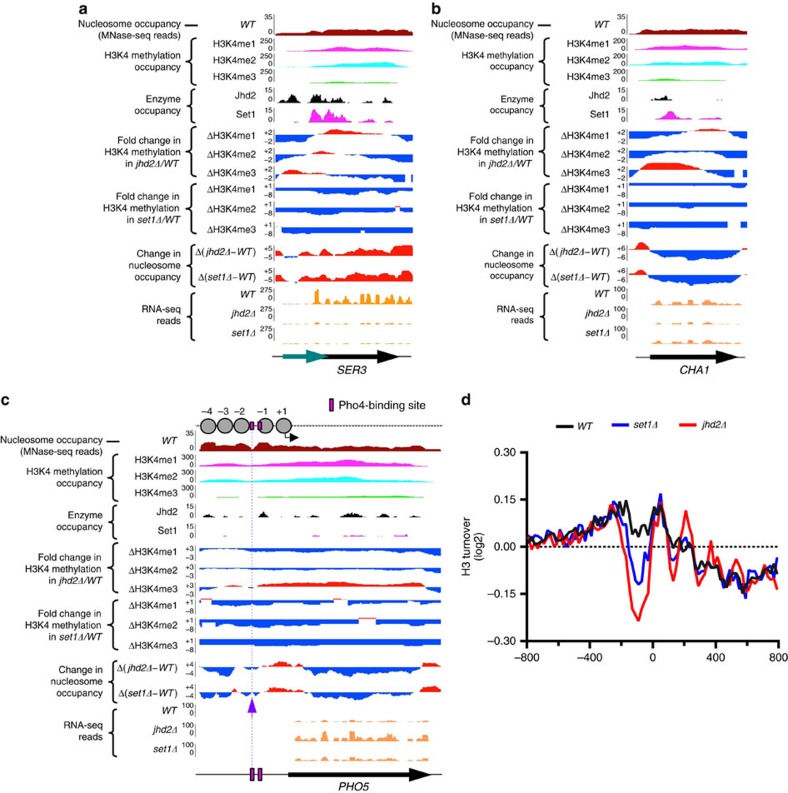
Co-regulation of nucleosomal occupancy and transcription by Set1 and Jhd2 at candidate target genes. (**a**–**c**) Changes to H3K4 methylation levels, nucleosomal occupancy and transcripts at *SER3*, *CHA1* and *PHO5* genes in *jhd2Δ* or *set1Δ* are shown. The normal nucleosomal, H3K4 methylation, Jhd2 and Set1 occupancies at target genes are also shown: reads for mononucleosomal DNA in WT shows native chromatin organization (brown). Reads for H3K4 monomethyl (me1, magenta), H3K4 dimethyl (me2, cyan) and H3K4 trimethyl (me3, green) marks in WT shows their distribution over candidate target genes. Enrichment values (qpois) for Jhd2 (black) and Set1 (pink) in WT show their occupancy at the indicated genes. Fold-change in the levels of H3K4me1, H3K4me2 and H3K4me3 marks in *jhd2Δ* or *set1Δ* relative to that in WT are shown. Fold-change in H3K4 methylation in *set1Δ* relative to WT was calculated using published datasets^42^. Increase or decrease in a given H3K4 methyl mark in the mutant is shown in red or blue, respectively. Mononucleosomal DNA from WT were subtracted from those obtained from *jhd2Δ* or *set1Δ* and visualized using a genome browser. Gain or loss in nucleosome occupancy in the mutant is represented in red or blue, respectively. RNA-seq reads showing transcript levels for the three target genes in *WT*, *jhd2Δ* or *set1Δ* strains are shown (orange). Teal arrow indicates *SRG1*, a gene coding for regulatory non-coding RNA involved in *SER3* regulation^34^. In **c**, schematic diagram at top shows the nucleosome organization at *PHO5*. Circles, well-positioned promoter nucleosomes; black arrow, TSS; and pink box, the Pho4 binding site, determined using published datasets^69^,^70^. Grape coloured arrow and dotted line indicate decrease in nucleosomal occupancy at the Pho4 binding site. (**d**) The average mean profile for histone H3 turnover across 800 bp region upstream (−) or downstream (+) of the TSSs (0) at all 236 yeast ribosome biogenesis or Ribi genes in *WT*, *set1Δ* and *jhd2Δ* are shown.

To further understand the role for Set1 and Jhd2 in gene regulation, we examined the specific changes at canonical target genes. Set1 and Jhd2 positively co-regulate *SER3* and *CHA1* expression (Fig. 2c). While nucleosomal occupancy is increased at *SER3* (Fig. 5a), it is dramatically reduced over *CHA1* coding region in *set1Δ* and *jhd2Δ* (Fig. 5b). Increased nucleosomal occupancy and an MNase-resistant chromatin structure correlate with the high nucleosomal turnover at the co-activated genes (Figs 4f and 5a,b), and indicate increased histone deposition or a stable nucleosome assembly that could block RNA Pol2 progression. In contrast, low nucleosomal occupancy at *CHA1*, as evidenced by increased MNase digestion, in spite of a high nucleosomal turnover indicates rapid cycles of nucleosome assembly and disassembly, which could also adversely affect RNA Pol2 progression. Thus, Set1 and Jhd2 activities might co-regulate nucleosome assembly and/or disassembly to promote transcriptional elongation at various co-activated target genes.

Transcriptional repression at *PHO5* is a result of well-positioned promoter nucleosomes that occlude the binding sites for transcriptional activator Pho4 (ref. 49), as confirmed in our WT nucleosome occupancy track (Fig. 5c, see MNase-seq reads). Nucleosome occupancy over Pho4 binding site is decreased at *PHO5* and *PHO89* promoters in *set1Δ* and *jhd2Δ* (Fig. 5c; Supplementary Fig. 13), which likely increases accessibility to Pho4 and augments transcription, as indeed observed in our gene expression studies (Fig. 1a–d). Set1 and Jhd2 also co-repress Ribi genes (Figs 1 and 2). Nucleosomal occupancies at candidate Ribi genes showed no apparent change other than that commensurate with increased transcription (Supplementary Fig. 14a,b). However, we found a severe reduction in promoter nucleosomal turnover at all 236 yeast Ribi genes^50^ in *set1Δ* and *jhd2Δ* (Fig. 5d; Supplementary Fig. 12c). Therefore, Set1 and Jhd2 co-regulate histone turnover to retain promoter nucleosomes at phosphate-responsive and Ribi genes to likely restrict transcriptional initiation.

### Set1 and Jhd2 co-regulate genome-wide chromatin dynamics

**Figure 6:**
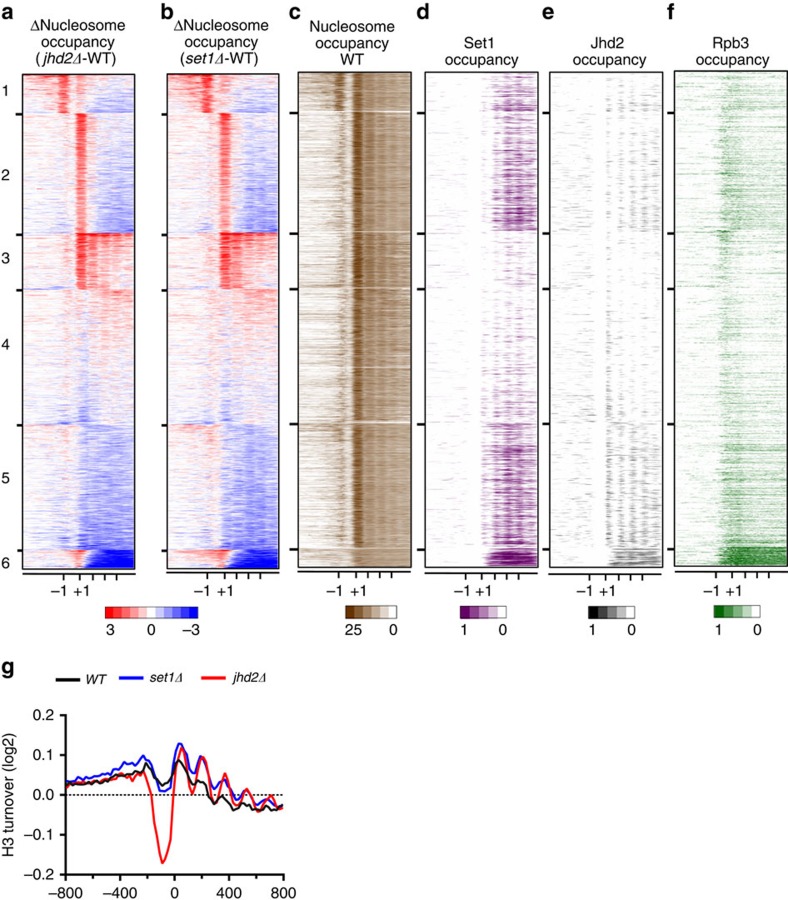
Set1 and Jhd2 co-regulate chromatin structure and nucleosomal turnover genome-wide during transcriptional regulation in yeast. (**a**) Nucleosome occupancy ratios (Δnucleosome occupancy) between *jhd2Δ* and control WT across all yeast genes are shown as a heat map, where gain or loss in nucleosome occupancy are in red and blue, respectively. Genes in rows are organized into six k-means clusters. Columns represent 50 bp windows over ±800 bp regions relative to the TSS. Relative positions for −1, +1 and other coding regions nucleosome are indicated at the bottom. Windows overlapping neighbouring genes were excluded. (**b**) Heat map for Δnucleosome occupancy between *set1Δ* and *WT* across all yeast genes. Heat map details are as described for **a**. (**c**) Native nucleosome occupancy for genes in the six *k*-means clusters generated from reads obtained in *WT* are shown. Nucleosome occupancy above global mean is in brown. (**d**,**e**) Heat maps for Set1 and Jhd2 occupancy within the six clusters are shown. (**f**) Heat map for Rpb3 occupancy within the six *k*-means clusters, using published data^51^, is shown. Pol2 subunit occupancy above global mean is in green (Supplementary Fig. 14b). (**g**) The average mean profile for histone H3 turnover across 800 bp region upstream (−) or downstream (+) of the TSSs (0) at all yeast genes in *WT*, *set1Δ* and *jhd2Δ* are shown.

We then mapped the nucleosomal occupancy and change relative to WT control for both *set1Δ* and *jhd2Δ* mutants at the TSSs across all yeast genes and grouped them using *k*-means clustering. Our analyses revealed distinct changes across all yeast genes (Fig. 6a–c), similar to that observed at the functional target genes (Fig. 4a,b). Importantly, changes are remarkably consistent between *jhd2Δ* and *set1Δ*, further establishing the co-regulation of chromatin structure by these opposing enzymes. ChIP-seq data showed genome-wide co-localization and co-regulation of H3K4 methylation dynamics by Set1 and Jhd2 at all six *k*-means clusters (Fig. 6d,e; Supplementary Figs 15 and 16). Clusters with high Set1 and Jhd2 occupancy showed a greater loss of nucleosomal occupancies in *jhd2Δ* and *set1Δ* (clusters 1–2 and 5–6, Fig. 6), further supporting the role for Set1 and Jhd2 in controlling chromatin structure. We also found a genome-wide co-regulation of nucleosomal turnover by Set1 and Jhd2 (Fig. 6g). Promoter nucleosomal turnover was severely reduced in *jhd2Δ* and to an extent in *set1Δ*, but nucleosomal turnover over coding regions was increased in both mutants (Fig. 6g). Collectively, these results revealed a remarkable genome-wide co-regulation of H3K4 methylation dynamics, nucleosomal turnover and chromatin structure by Set1 and Jhd2.

We then asked whether the distinct chromatin effects co-regulated by Set1 and Jhd2 are correlated with transcriptional activities of genes present in each of the six clusters. We surveyed the distribution of two Pol2 subunits, Rpb3 and Rpo21 (ref. 51), across each gene and ranked the genes in the same order as the nucleosome occupancy data (Fig. 6a,b) or as a mean cluster profile plot (Supplementary Fig. 17b). We determined the transcriptional frequency of genes in each cluster by intersecting them with groups of yeast genes categorized into five classes of increasing transcriptional rate (mRNA/h)^52^ (Fig. 6). Genes showing statistically significant intersection in these comparisons were subjected to GO analysis, which showed correlations between co-regulation of chromatin structure by Set1 and Jhd2 and transcriptional regulation at genes controlling specific biological process (Fig. 6h; Supplementary Data 4). We also examined changes to H3K4 methylation levels and nucleosomal turnover at these clusters in *set1Δ* and *jhd2Δ* strains. Our findings from these analyses are described below.

Set1 and Jhd2 regulate ribosomal protein and Ribi genes^21^,^26^,^27^,^53^ (Figs 1e and 5d), which are present within clusters 1 and 2, respectively. At clusters 1–2, Set1 and Jhd2 destabilize either one or both flanking nucleosomes while increasing histone turnover to retain promoter nucleosomes only in cluster 2 (Fig. 6a–c; Supplementary Figs 18 and 19). Cluster 3 contains the poorly transcribed genes with an apparently ‘stalled' Pol2 at the promoter (Table 3; Supplementary Fig. 17b). Set1 and Jhd2 co-regulate nucleosome removal at these ‘silent' genes in a histone turnover-independent mechanism (Fig. 6a,b; Supplementary Figs 18 and 19). At transcribed genes in clusters 4 and 5, Set1 and Jhd2 decrease histone turnover over coding regions (Supplementary Figs 18 and 19), and they decrease nucleosomal occupancy at cluster 4, but increase it at cluster 5 (Fig. 6a,b). Therefore, Set1 and Jhd2 co-regulate nucleosome disassembly and assembly at clusters 4 and 5, respectively, akin to the *SER3* and *CHA1* genes (Fig. 5a,b). Cluster 6 contains very high Pol2, Jhd2 and Set1 occupancies, a very high rate of histone turnover over coding regions and a distinct absence of an organized chromatin structure (Fig. 6c–f; Supplementary Figs 18 and 19), all of which correlate well with the high rate of transcription (Table 3). At cluster 6, Set1 and Jhd2 decrease +1 nucleosomal occupancy, but increase nucleosomal occupancy while decreasing histone turnover over coding regions (Fig. 6a,b; Supplementary Figs 18 and 19). Thus, Set1 and Jhd2 appear to co-regulate nucleosomes differently at different regions within highly transcribed genes. While Set1 and Jhd2 disrupt the +1 nucleosome around TSSs, they stabilize coding region nucleosomes during transcriptional elongation. Therefore, Set1 and Jhd2 co-regulate chromatin dynamics in distinct ways at different groups of yeast genes and at different regions within a gene.

## Discussion

Roles for Set1 in positive or negative regulation of transcription are known^5^,^12^,^17^,^21^,^54^. A positive or negative role for Jhd2 in protein-coding gene transcription^26^, while acting as a repressor of intergenic non-coding transcription during yeast gametogenesis^27^, is known. Here, we report that Set1 and Jhd2, two chromatin modifiers with opposing enzymatic functions, act in synergy at the same target genes: loss of one results in the same transcriptional response as the loss of the other at many yeast genes, implying a complex function of chromatin biology centred around maintaining the proper balance of histone marks by two opposing enzymes. Through a series of targeted and genome-wide experiments, we show that the chromatin environment helps to dictate the transcriptional response upon the loss of either enzyme. Genes repressed by the loss of Set1/Jhd2 have a chromatin signature distinct from genes that are activated by the loss of Set1/Jhd2. Part of this signature and response is the control of nucleosome occupancy and histone turnover, implying the role of H3K4 methylation in these activities and ultimately in transcriptional regulation. In summary, our study reveals a novel mechanism wherein combined activities of Set1 and Jhd2, via H3K4 methylation and demethylation, modulate nucleosomal turnover and chromatin dynamics during transcriptional regulation.

**Figure 7:**
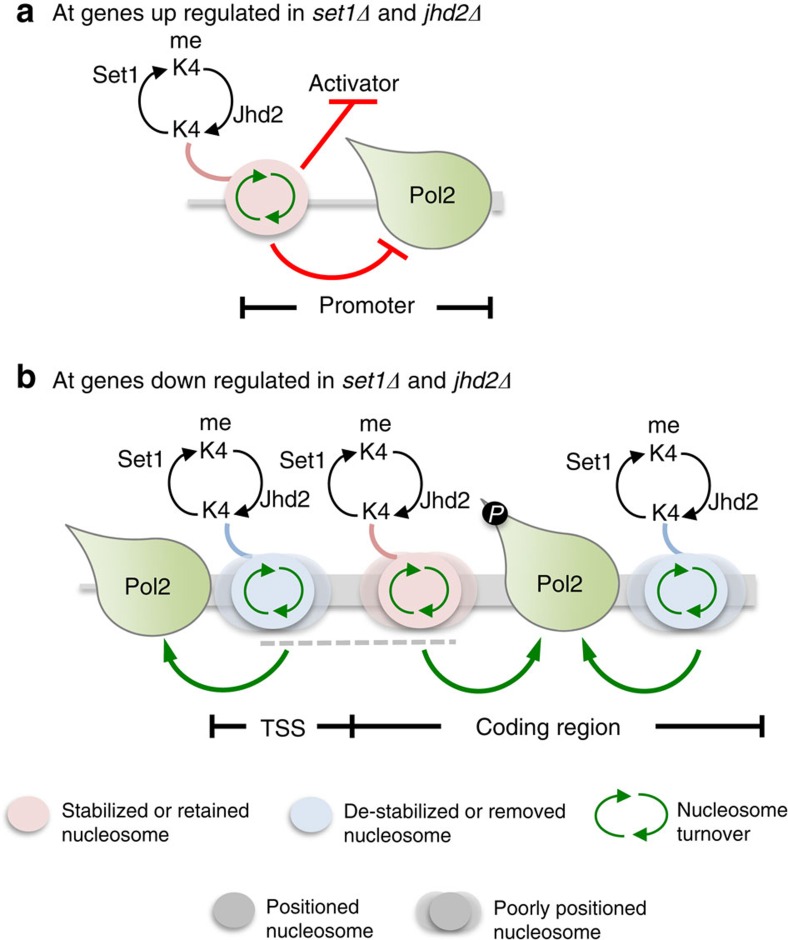
Model for Set1 and Jhd2-mediated co-regulation of chromatin structure and RNA Pol2 functions. (**a**) Target genes co-repressed by Set1 and Jhd2 are packaged into a well-defined chromatin organization. At these genes, Set1 and Jhd2 activities via H3K4 methylation–demethylation impact histone turnover at promoters leading to nucleosome retention, which restricts activator and/or RNA Pol2 binding. Thus, Set1 and Jhd2 are proposed to primarily co-regulate transcription initiation at these target genes. (**b**) Target genes co-activated by Set1 and Jhd2 are present in a poorly organized chromatin configuration, contain high levels of H3K4me2 and undergo high nucleosomal turnover over coding region. Set1 and Jhd2 activities via modulating H3K4 methylation levels impact nucleosomal turnover over coding regions, and proposed to remove +1 nucleosomes in front of RNA Pol2 and stabilize or retain nucleosomes behind the elongating RNA Pol2. Thus, Set1 and Jhd2 are proposed to co-regulate transcription elongation at these target genes. *P*, phosphorylated form of RNA Pol2 engaged in elongation.

Here, we have shown that Set1 and Jhd2 co-repress transcription at *PHO5* and other yeast genes including Ribi genes. Our study shows that transcriptional co-repression occurs through combined action of Set1 and Jhd2 on promoter nucleosomal turnover, which allows nucleosome retention and inhibition of activator and/or RNA Pol2 binding (Fig. 7a). The Rpd3L histone deacetylase (HDAC) complex is linked to transcriptional repression at *PHO5* and Ribi genes^21^,^55^,^56^. Rpd3L complex subunits, Pho23 and Cti6, contain PHD domain that binds H3K4 methyl marks^57^. Rpd3 was also demonstrated to stabilize nucleosomes *in vivo* independent of its catalytic activity^58^. Therefore, one possibility is that combined Set1 and Jhd2 activities might modify promoter nucleosomes in a manner that facilitates association and/or action of HDAC complexes, such as, Rpd3L, resulting in reduced nucleosomal turnover and a repressive nucleosome formation. Chromatin remodelers, including RSC, are known to eject promoter nucleosomes^59^. RSC chromatin remodeling complex is required for *PHO5* promoter opening^60^, and Set1 and Jhd2 may regulate remodeler activity through modulation of H3K4 methylation.

Our studies show that Set1 and Jhd2 control nucleosomes over TSSs and/or coding regions to positively co-regulate transcription at certain target genes. Target genes under positive co-regulation are characterized by high H3K4me2 and H3K4me1 levels, possess high nucleosomal turnover across coding regions and packaged in a disorganized chromatin structure (Figs 3 and 4). One could postulate that Set1 and Jhd2 via maintaining H3K4me1-H3K4me2 levels control nucleosomal turnover over coding regions and regulate RNA Pol2 progression at these genes. We further postulate that Set1 and Jhd2 could co-regulate either nucleosomal assembly or disassembly at different target genes and at different regions within a gene, as in the case of *SER3* and *CHA1* genes (Fig. 5a,b) or genes in clusters 4–6 (Fig. 6). On the basis of our findings, we propose that Set1 and Jhd2 via H3K4 methylation–demethylation destabilize the +1 nucleosome likely to permit RNA Pol2 entry into the coding region (that is, regulation of initiation–elongation transition), whereas they stabilize or destabilize coding region nucleosomes to likely regulate RNA Pol2 progression during transcriptional elongation (Fig. 7b).

H3K4me3 is implicated in positive as well as negative transcriptional regulation^21^,^61^. H3K4me3 is remarkably enriched at the 5′-ends of vast majority of transcriptionally active as well as ‘silent' yeast genes (Supplementary Fig. 15). Thus, one possibility is that Set1 and Jhd2 might regulate nucleosomal turnover through targeting H3K4me3 levels during transcriptional co-regulation. H3K4me2 was shown to recruit Set3 HDAC complex to 5′-end of genes^4^ and is implicated in transcriptional fidelity^6^. A high level of H3K4me2 and H3K4me1 marks compared to H3K4me3 occurs at Set1 and Jhd2 co-activated target genes (Fig. 7b). Assuming H3K4me1 is a transitional state, one could propose H3K4me2 as the transcriptional activation mark at co-activated genes. Intriguingly, deletion of either *SET1* or *JHD2* leads to a genome-wide reduction in H3K4me2 mark (Supplementary Fig. 15a,b), which further implicates H3K4me2 as a key regulatory signal for nucleosomal turnover.

How might Set1 and Jhd2 via H3K4 methylation–demethylation control chromatin dynamics? A role for the H3 N-terminal tail in nucleosome stabilization was predicted using computational modelling^62^. Thus, one possibility is that addition or removal of methyl groups from the K4 residue in H3 N-terminal tail might stabilize or destabilize intra-nucleosomal interactions. Alternatively, Set1 and Jhd2 via H3K4 methylation–demethylation might regulate the functions of many chromatin modifying or remodeling complexes with an ability to alter histone–histone or histone–DNA interactions within a nucleosome, particularly those containing subunits with ‘reader' domains that bind modified or unmodified H3K4 residue. We propose two models: one, a sequential dual state model, where Set1 and Jhd2 act sequentially on the two K4 residues present in a nucleosome and sustain a methylation–demethylation cycle, which then signals nucleosomal retention or removal. In the second model, Set1 and Jhd2 might differentially target the two K4 residues to establish an asymmetrically modified nucleosome, which could act as a composite mark recognized by multiple ‘reader' modules present in chromatin modifying or remodeling complexes. Future investigations will discern which one of these scenarios underlies the Set1 and Jhd2-mediated co-regulation of nucleosomal turnover and chromatin dynamics.

## Methods

### DNA constructs

Plasmid p414ADHFlag was created by PCR amplifying a sequence coding for the Flag epitope, digesting it with Xba1 and Spe1 before ligation downstream of the *ADH1* promoter in vector p414ADH^63^. Plasmid p414ADHFlagSET1 was obtained by gap-repair approach in yeast; PCR products coding for Set1 N-terminal (amino acids 1–355) and C-terminal regions (amino acids 762–1080) were mobilized sequentially downstream of the Flag epitope in p414ADHFlag, linearized with PstI and transformed into yeast. PCR-based mutagenesis of the *SET1* 3′ORF was performed to create *SET1-G990E*. The gap-repair approach was used to then obtain p414ADHFlagSET1G990E. Plasmid to express catalytic-dead mutant of Set1 (set1-C1019A) in vector p426MET25 was described previously^38^. A fragment of the *SET1* 3′ORF region containing the mutation coding for C1019A was excised and used to replace the WT *SET1* sequence in p414ADHFlagSET1. To express WT Jhd2 or its catalytic-dead mutant (jhd2-H427A), the *JHD2-9myc* or *jhd2-H427A-9myc* ORF regions were mobilized into p424ADH from constructs described previously^22^. The *ADH1* promoter, coding sequence for WT or mutant *SET1* or *JHD2* and the *CYC1* transcription termination sequence were excised using KpnI and SacI from constructs described above, and mobilized into the same restriction sites in yeast integration vector pRS306 (ref. 64).

Plasmid to express N terminally V5 epitope-tagged Set1 was created as follows: plasmid p9Myc*SET1-LEU2* (ref. 17) (kindly provided by Dr Vincent Géli) was digested with HindIII and EcoRI. The resulting fragment was ligated to HindIII and EcoRI digested pRS306 to yield p9Myc*SET1-URA3*. Next, gBlocks gene fragment (Integrated DNA Technologies, IDT) was synthesized to contain sequence for eight copies of the V5 epitope tag flanked by 20 bp of promoter or coding region present immediately adjacent to *SET1* start codon in p9Myc*SET1-URA3*. A fragment of the *SET1* coding region also was amplified containing 20 bp homology to the 3′-end of the synthesized gene fragment and 20 bp homology to the region downstream of the SnaB1 site in *SET1* coding region at its 5′- and 3′-ends, respectively. The gene fragment and the PCR product were co-mobilized into Not1 and SnaB1 digested p9Myc*SET1-URA3* using sequence and ligation-independent cloning in order to obtain plasmid p8V5*SET1-URA3*. To create plasmid pMC001 containing the V5 to 2XFlag RITE cassette, the HA-6XHIS epitope tag in plasmid pMT004 (ref. 65) (a gift from Fred van Leeuwen, Addgene plasmid # 64759) was replaced with an IDT gBlocks gene fragment coding for two copies of the Flag epitope tag.

### Yeast strains and media

Yeast were grown in YPAD broth (1% yeast extract, 2% peptone, 2% dextrose and 0.004% adenine hemisulfate) at 30 °C to an OD A_600_ (optical density at absorbance 600 nm) of 0.8–1.0.

Detailed genotypes of yeast strains used in this study are listed in Supplementary Table 1. Strain HTABWT was made by transforming parental strain FY406 with a plasmid (*HTA1-HTB1*, *CEN*, *HIS3*) and subsequent plating on a medium containing 5-fluoroorotic acid to cure pSAB6. HTABWT serves as the WT control strain and the founder strain in this study. Genomic DNA isolated from *jhd2Δ* (Open Biosystems) was used as the template to PCR amplify 500 bp region upstream or downstream of the *JHD2* ORF replaced with the *KanMX4* cassette. Amplified PCR product was transformed into FY406 to replace the *JHD2* gene with the *KanMX4* cassette to obtain the deletion mutant (YZS652). Strain YMC70 was then obtained by transforming plasmid *HTA1-HTB1*, *CEN*, *HIS3* into YZS652 and shuffling out pSAB6 using 5-fluoroorotic acid^26^. A fragment containing 500 bp regions upstream and downstream of *SET1* ORF replaced by the *KanMX4* cassette was PCR amplified using genomic DNA isolated from YZS515, transformed into HTABWT to delete the *SET1* gene and obtain mutant strain YMC64 (ref. 26). The *NatMX4* cassette along with ∼40 bp sequence homologous to the *JHD2* promoter and 3′ terminator regions was amplified using vector pAG25 as the template, and transformed into YMC64 to create the double mutant strain YMC65. To overexpress Set1, Set1-G990E or Jhd2, plasmids described above harbouring *Flag-SET1*, *Flag-SET1-G990E* or *JHD2-9myc* driven by the *ADH1* promoter in pRS306 were linearized with Stu1 and integrated into the *URA3* locus in strain HTABWT. To express the catalytic-dead versions of Set1 or Jhd2, plasmids with *ADH1* promoter driven Flag-set1-C1019A or jhd2-H427A-9myc in pRS306 were digested with Stu1 and integrated into the *URA3* locus in YMC64 and YMC70, respectively. p8V5*SET1::URA3* was digested with SnaB1 and transformed into HTABWT to yield strain YMC127 expressing V5 epitope-tagged version of Set1.

Strains to measure histone H3 turnover were created as follows. YMC120 expressing the Cre recombinase was obtained by integrating MluI-digested pSS146 (ref. 65) (Addgene # 66740) into HTABWT. Strain YMC122 expressing histone H3 tagged with the RITE cassette (V5-LoxP-HphMX-LoxP-2xFlag) was then obtained by transforming YMC120 with a PCR product amplified using pMC001 as template and containing 40 bp sequence homology to either side of the stop codon in *HHT1* gene. To avoid interference from non-tagged H3 and to maintain the H3-H4 stoichiometry, the other gene coding for histone H3 (*HHT2*) and that coding for histone H4 (*HHF2*) were replaced with a *NatMX4* cassette in YMC122 in order to obtain strain YMC124. *SET1* or *JHD2* ORF was then replaced in YMC124 with the *KanMX4* cassette to obtain deletion mutants YMC125 and YMC128, respectively.

### Western blotting

To examine H3K4 methylation or epitope-tagged H3, cell extracts were prepared following the bead-beat procedure as described^66^. A twofold serial dilution of extracts were resolved in 15% SDS–PAGE and transferred onto a polyvinylidene difluoride (PVDF) membrane. The following antibodies were used in western blotting (dilutions used, source and catalogue numbers are indicated within parentheses): α-H3K4me1 (1:1,000, Active Motif, 39,297), α-H3K4me2 (1:10,000, Millipore, 07–030), α-H3K4me3 (1:7,500, Active Motif, 39,159), α-V5 (1:50,000, Bio-Rad, MCA1360) and α-Flag (1:5,000, Sigma, F3165). The histone H3 loading was monitored using α-H3 (1:2,000, Abcam, ab46765). Full-length images of the blots are shown in Supplementary Figs 20–22.

### Strand-specific reverse transcribed (RT)-qPCR

Yeast cells (10^7^) in mid-log phase were collected for total RNA extraction using the MasterPure yeast RNA purification kit (Epicentre). RNA was then digested with TURBO DNA-free (Life Technologies) to eliminate contaminating DNA and purified using RNeasy Kit (Qiagen). RNA concentration was measured using NanoDrop 2000 UV–vis Spectrophotometer (Thermo Scientific). To obtain sense or antisense strand-specific complementary DNA, total RNA (1–2 μg) was RT using the Transcriptor first-strand complementary DNA synthesis kit (Roche) and a reverse strand primer listed in Supplementary Table 2. To measure *SER3* and *CHA1* transcript levels, total RNA was reverse transcribed using oligo-dT primer. Transcript levels were assessed using qPCR in a Bio-Rad CFX Connect Real-Time PCR Detection System employing both forward and reverse primers (Supplementary Table 2). Fold-change in sense or antisense transcript level in a mutant was calculated using the 2^−ΔΔCT^ method relative to the respective transcript level in the WT control. Four independent biological replicates and triplicate PCR were performed.

### RNA-seq

Three independent cultures for strains HTABWT, YMC42, YMC43, YMC64 and YMC70 were grown to mid-log phase. A total of 3 × 10^8^ cells per culture were collected and washed with nuclease-free water (Sigma). Total RNA was isolated using RiboPure-Yeast Kit (Life Technologies). To eliminate residual DNA, total RNA was treated with 2 μl DNase I (TURBO DNA-free Kit; Life Technologies) for 45 min at 37 °C. RNA was digested once more using DNase I (RNase-Free DNase set; Qiagen) for 10 min at room temperature before purification using the RNeasy Kit (Qiagen). Before the library construction for RNA-seq, the quality of RNA was ascertained using Bioanalyzer (Agilent), and the RNA integrity number (RIN) for the samples ranged from 6.8 to 8.1. The Ribo-Zero Magnetic Gold Kit (Yeast) was used to remove ribosomal RNA from total RNA used for stranded RNA-seq. Library construction was performed using the Illumina TruSeq RNA Sample Preparation Kit version 2 and sequenced using the Illumina Hiseq2000 sequencer.

### ChIP-seq

Overall, 500 ml cultures each for the no-tag control (HTABWT), Jhd2-12V5 (YMC85) and 8V5-Set1 (YMC127) strains were grown to mid-log phase and cross-linked with 1% formaldehyde for 20 min at room temperature. Crosslinking was stopped using 125 mM glycine. Cells were washed extensively with 1 × phosphate-buffered saline (PBS). Four sets of 100 × 10^7^ cross-linked cells per strain were collected and stored at −80 °C. Cells were thawed, converted to spheroplasts using Lyticase (250 units, Sigma) and lysed with 0.25% Triton X-100 before isolating crude nuclei using a sucrose cushion. Nuclei were washed with micrococcal nuclease (MNase) digestion buffer (25 mM Tris–HCl pH 7.5, 50 mM NaCl, 5 mM MgCl_2_, 1 mM CaCl_2_) and resuspended in the same buffer before digesting chromatin with 20 units of MNase (Worthington Biochemicals) for 15 min at 37 °C. Digestion was stopped by adding 10 × stop buffer (50 mM Tris–HCl pH 7.5, 500 mM NaCl, 100 mM EDTA, 20 mM EGTA, 10% Triton X-100) and clarified by high-speed centrifugation (16,100*g* for 10 min at 4 °C). Clarified supernatants for each strain obtained from four sets of cross-linked cells were pooled together (∼4.5 ml).Overall, 50 μl was set aside for isolating control input DNA. The remaining soluble chromatin was split equally into four tubes (1.3 ml each) and pre-cleared using 25 μl blocked Dynabeads Protein G for 1 h at 4 °C with end-over-end rotation. Blocking of magnetic beads was performed at 4 °C with end-over-end rotation for >24 h using a solution made up of FA buffer (50 mM HEPES-KOH, pH 7.5, 140 mM NaCl, 1 mM EDTA, 1% Triton X-100, 0.1% sodium deoxycholate), 140 mM NaCl, bovine serum albumin (0.2 mg ml^−1^, New England Biolabs) and fish skin gelatin (0.5%, Sigma). Blocked beads were washed with binding buffer (MNase digestion buffer+1 × stop buffer) before use. A total of 15 μl α-V5 antibody (Bio-Rad, Catalogue No. MCA1360) was then added to pre-cleared chromatin and incubation was performed overnight at 4 °C with end-over-end rotation. And 50 μl blocked Dynabeads Protein G was added to capture the antigen–antibody complexes and incubation was continued for an additional 2.5 h. Beads were then sequentially washed 5 min each with end-over-end rotation at 4 °C as follows: three washes with FA buffer+140 mM NaCl, two washes with FA buffer+1000, mM NaCl, twice with FA buffer+500 mM NaCl, and one wash each with LiCl/NP40 buffer (10 mM Tris–HCl, pH 8.0, 250 mM LiCl, 0.5% NP40, 0.5% sodium deoxycholate) and TE (10 mM Tris–HCl, pH 8.0, 1 mM EDTA). Elution, reverse crosslinking of ChIP and input DNA, RNase A and Proteinase K digestion steps were performed as described^66^. ChIP and input DNA were purified using Qiagen MinElute PCR Purification kit following manufacturer's protocol except two washes each of buffer QG and PE were performed. ChIP enrichment was examined by qPCR using SYBR green, which showed >100-fold enrichment for 8V5-Set1 and Jhd2-12V5 at the *PMA1* 5' region relative to the no-tag control. Library construction was performed using NEBNext Multiplex Oligos (Index Primers Set 1) and NEBNext ChIP-seq Library Prep Reagent Set for Illumina and sequenced using the Illumina Hiseq2000 sequencer.

### qChIP-seq

Control (HTABWT), Flag-SET1-G990E (YMC43) and *jhd2Δ* (YMC70) strains were grown to mid-log phase and cross-linked with 1% formaldehyde for 20 min at room temperature. Crosslinking was stopped using 0.125 mM glycine and cells were washed with 1 × PBS. Multiple aliquots of 40 × 10^7^ cross-linked cells each were prepared. *C. glabrata* (Anderson) Meyer et Yarrow Strain Designation: NCYC 388 (ATCC 36909) was grown at 25 °C (ref. 39) and subjected to formaldehyde crosslinking as described for budding yeast, and individual aliquots containing 20 × 10^7^ cells each were collected. Cross-linked *S. cerevisiae* (40 × 10^7^) and *C. glabrata* (20 × 10^7^) cells were mixed together in FA140+SDS buffer (50 mM HEPES-KOH, pH 7.5, 140 mM NaCl, 1 mM EDTA, 1% Triton X-100, 0.1% sodium deoxycholate, 140 mM NaCl, 0.1% SDS) and lysed by bead beating. Lysates from two sets of *S. cerevisiae* and *C. glabrata* admixture (that is, 120 × 10^7^ cells in total) were pooled and sonicated in a Diagenode Bioruptor for 30 min (6 cycles of 5 min each, 30 s ON and 30 s OFF at high setting). Soluble chromatin was obtained after two sequential high-speed centrifugations of the sonicated lysate (16,100*g* for 5 min and 15 min). SDS was removed by dialysis as follows. Soluble chromatin obtained from four batches of control or mutant budding yeast and candida admixture were pooled, transferred to a 5 ml Float-A-Lyzer G2 dialysis device (3.5–5 kD MWCO) (Spectra/Por) and dialyzed initially for 2 h in 1 l FA140 buffer followed by an overnight dialysis in fresh 4 l FA140 buffer. Dialysate was centrifuged at 16,100*g* for 15 min at 4 °C. A 100 μl aliquot was set aside for isolating input DNA. The remaining dialysate was split equally into three tubes and pre-cleared using 50 μl blocked Dynabeads Protein A for 1 h at 4 °C with end-over-end rotation. Dynabeads Protein A were blocked with bovine serum albumin and fish skin gelatin as described above except bead blocking was done for 4 h. Pre-cleared lysate was combined with either α-H3K4me1 (20 μl, Active Motif) or α-H3K4me2 (10 μl, Millipore) or α-H3K4me3 (10 μl, Active Motif) antibody and 100 μl blocked Dynabeads Protein A, and incubated overnight at 4 °C with end-over-end rotation. Beads were initially washed with three times with FA140 buffer by inversion followed by sequential washes (5 min each with end-over-end rotation): FA140 buffer (2 times), FA1000 buffer (2 times), FA500 (2 times), LiCl/NP40 buffer (1 time) and TE (1 time). Elution of ChIP DNA, reverse crosslinking and purification of ChIP and input DNA, library preparation and sequencing were performed as described above. Two independent biological replicates were performed.

### Recombination-induced tag exchange (RITE)

YMC124, YMC125 and YMC128 were grown overnight in 125 ml YPAD medium containing hygromycin (200 μg ml^−1^).Overall, 40 × 10^7^ cells were set aside for western blotting of pre-switch cells. Overnight cultures were diluted 1:10 into fresh 270 ml YPAD medium and grown for 30 h. Cells were then grown overnight in the presence of 2 μM β-estradiol (E-8875, Sigma) to induce tag switch. And 40 ml overnight culture for each strain was diluted into 310 ml fresh YPAD medium. Cells were arrested in G1 phase by growing them for 4 h in the presence of α-factor (5 ng μl^−1^, Zymo Research). G1 cells from each strain were subjected to formaldehyde crosslinking and also collected for western blotting.

### ChIP-nexus

Cross-linked G1 cells for strains YMC124, YMC125 and YMC128 were lysed, sonicated and immunoprecipitated with α-V5 or α-Flag M2 (Sigma) antibody as described above for qChIP-seq. ChIP-nexus library preparation was performed following the procedure described by Zeitlinger and colleagues^48^. Briefly, ChIP DNA on beads was washed sequentially with buffer A (10 mM TE, 0.1% Triton X-100), buffer B (20 mM Tris–HCl pH 8.0, 150 mM NaCl, 5 mM EDTA, 5.2% sucrose, 1.0% Triton X-100, 0.2% SDS), buffer C (5 mM Tris–HCl pH 8.0, 250 mM NaCl, 25 mM HEPES-KOH pH 7.5, 0.5% Triton X-100, 0.05% sodium deoxycholate, 0.5 mM EDTA) and buffer D (10 mM Tris–HCl pH 8.0, 250 mM LiCl, 0.5% NP40, 0.5% sodium deoxycholate, 10 mM EDTA). Bead-bound ChIP DNA was subjected to end repair, dA-tailing, Nexus adaptor ligation, 5′ overhang fill-in, 5′ end trim, λ exonuclease and RecJ f exonuclease digestions. Beads were washed with buffers A–D and equilibrated in 10 mM Tris–HCl, whose pH was same as that of the buffer used in the next enzymatic step. Finally, beads were washed three times with RIPA buffer (50 mM HEPES pH 7.5, 1 mM EDTA, 0.7% sodium deoxycholate, 1% NP40, 0.5 M LiCl). ChIP DNA was extracted from beads using an elution buffer (50 mM Tris–HCl pH 8.0, 10 mM EDTA, 1% SDS) and incubation at 65 °C in a Thermomixer with shaking. After addition of equal volume of TE, reversal of crosslinking was performed at 65 °C for 6 h followed by RNase A and Proteinase K digestion. ChIP DNA was subjected to phenol and phenol–chloroform extractions before addition of glycogen (10 μg μl^−1^) and ethanol precipitation. Purified DNA was dissolved in 12 μl water, and 1 μl was used in qPCR to check ChIP efficiency at the *PMA1* 5′ region. The remaining 11 μl ChIP DNA was denatured at 95 °C for 5 min followed by single-strand DNA circularization using CircLigase (Epicentre) at 60 °C for 1 h. The cut oligo was then annealed to the circularized single-strand DNA in a Thermocycler (95 °C for 1 min, 25 °C for 1 min (1% ramp), 25 °C for 30 min), linearized using FastDigest BamHI (Fisher Scientific) before ethanol precipitation. ChIP DNA libraries from control and mutant strains were PCR amplified using universal and bar-coded primers present in the NEBNext Multiplex Oligos Index Primers Set 1. Library DNA was gel purified using Qiagen MinElute Gel Extraction kit and subjected to single end sequencing in Illumina Hiseq2000 sequencer. Two independent biological replicates were performed.

### MNase-seq

Three independent mid-log phase cultures (OD A_600_ of 0.8–1.00) for HTABWT, YMC64 (*set1Δ*) and YMC70 (*jhd2Δ*) were cross-linked with 1% formaldehyde as described^66^. A total of 33 × 10^7^ cells per culture were collected, washed twice with 1 × PBS and once with spheroplasting buffer (1M Sorbitol, 10 mM Tris–HCl pH 7.5, 15 mM 2-mercaptoethanol and protease inhibitors (PIs): 1 mM PMSF, 1 μg ml^−1^ aprotinin, 1 μg ml^−1^ leupeptin, 1 μg ml^−1^ pepstatin A). Cross-linked cells were resuspended in 1 ml fresh spheroplasting buffer before adding Lyticase (500 units; Sigma) and incubation in a 37 °C water-bath shaker for 15–20 min with gentle agitation. Spheroplasts were washed twice with 25 ml wash buffer (50 mM Tris pH 7.5, 100 mM KCl, 2.5 mM MgCl_2_, 400 mM Sorbitol and PIs), resuspended in MNase digestion buffer (50mm Tris pH 7.5, 100 mM NaCl, 5 mM MgCl_2_, 1 mM CaCl_2_ and 0.1% Triton X-100 and PIs) and digested with 10 units of MNase (Micrococcal Nuclease, Worthington Biomedical) at 37 °C for 15 min. Digestion was stopped by adding 100 μl 10 × stop buffer (50 mM Tris–HCl pH 7.5, 500 mM NaCl, 100 mM EDTA, 20 mM EGTA, 10% Triton X-100 and 1% sodium deoxycholate). After centrifugation, soluble chromatin (500 μl) was mixed with an equal amount of elution solution (1% SDS, 100 mM sodium bicarbonate) and NaCl (200 mM final concentration), reverse cross-linked for 5 h at 65 °C and ethanol precipitated overnight at −20 °C. After centrifugation, the DNA pellet was washed with cold 70% ethanol, air-dried, dissolved in nuclease-free water. RNase A (2 μl of 10 mg ml^−1^ stock, Qiagen) digestion was performed at 37 °C for 30 min followed by Proteinase K digestion (2 μl, Roche) for 1 h at 42 °C in the presence 1 × Proteinase K digestion buffer (10 mM Tris–HCl pH 8.0, 5 mM EDTA, 0.5% SDS). DNA was purified using Qiagen PCR purification kit and eluted in 50 μl nuclease-free water. DNA concentration was measured using NanoDrop 2000 UV–vis Spectrophotometer (Thermo Scientific). An aliquot was resolved in 2.0% agarose gel and stained with ethidium bromide to check the extent of MNase digestion (supplementary Fig. 5). Equal amount of purified DNA (10 ng) was subjected to library construction using the NEBNext ChIP-seq Library Prep Reagent Set for Illumina and resolved in an 1.5% agarose gel. Adaptor-ligated and index primer-containing amplified DNA corresponding to mononucleosomes was purified using the Qiagen Gel Extraction Kit essentially following the manufacturer's protocol, except incubation to melt the gel was performed at 37 °C for 30 min. The library was then subjected to 50 bp single read sequencing in an Illumina Hiseq2000 sequencer.

### Bioinformatics analysis

Illumina sequencing reads were aligned to the *S. cerevisiae* genome (SGD release R64, UCSC SacCer3) using Novocraft novoalign software. For RNA-seq, the reads were aligned using an index to both the genome and known splice junctions, and splice junction alignments were converted into genomic coordinates. For the reference genome, reads were aligned to the *C. glabrata* genome (Ensembl Fungi release 30, GCA_000002545.2). MNase-seq and ChIP-seq alignments were filtered for a minimum mapping quality of 13. In most cases, with the exception of RNA-seq analysis, alignments from biological replicates were merged after the replicates were deemed to be sufficiently correlative. Replicates were compared by generating a raw alignment coverage track and performing a Pearson correlation between them using the utility bigWigCorrelate (http://hgdownload.soe.ucsc.edu/admin/exe/).

RNA-seq analysis was performed using the USeq package (http://useq.sourceforge.net), and multiple-replica differential gene expression analysis was performed using the R package DESeq (http://genomebiology.com/2010/11/10/R106). Genes passing two thresholds, an FDR of <5% and absolute log_2_ ratio of 0.585 (that is, 1.5-fold change), were considered differentially expressed and used in subsequent analysis. Normalized-read coverage was used for visualization in a genome browser to confirm changes in expression. Gene ontologies were determined using the Gene Ontology Term Finder available at the Saccharomyces Genome Database. Total counts, gene lists and other relevant metrics associated with the RNA-seq analysis are included in the Supplemental Tables 1 and 2.

MNase-seq and ChIP-seq analysis was peformed using the MACS version 2 (ref. 67) and BioToolBox (https://github.com/tjparnell/biotoolbox) packages. Nucleosome fragment coverage was generated by extending the reads to 148 bp and normalizing to read depth (reads per million) using BioToolBox bam2wig. Nucleosome difference maps were generated by macs2 bdgcmp by subtracting the WT nucleosome coverage from the mutant nucleosome coverage. ChIP enrichment was calculated using macs2 callpeak (keeping all duplicates, small and large lambda turned off) and macs2 bdgcmp to create fold enrichment and *q*-value tracks.

qChIP-seq analysis to calculate normalized H3K4me occupancy relative to a reference genome was performed as described^39^. Reads were first aligned to the reference *C. glabrata* genome. Unaligned reads were subsequently mapped to *S. cerevisiae*. Reads aligned to *C. glabrata* were also aligned to *S. cerevisiae* to determine the number of orthologous alignments. Depth-normalized fragment coverage was generated with BioToolBox bam2wig with an extension of 200 bp. These coverage tracks were further scaled for calibrated read depth by multiplying the coverage values with a calibration factor derived from the ratio of alignment numbers to the target and reference genomes following the formula (Input_Cg_ × ChIP_Sc_) per (Input_Sc_ × ChIP_Cg_), where Cg notation represents non-orthologous reads aligned to *C. glabrata* and Sc notation represents non-orthologous reads aligned to *S. cerevisiae*. To generate fold-enrichment tracks, genomic intervals with zero coverage were artificially set to a non-zero value of 1 (a value well below the genomic mean) and both fold enrichment and statistical *q*-value tracks were calculated with macs2 bdgcmp. Since both enrichment and depletion can occur, the *q*-value tracks were calculated in both directions, and the maximum value reported. Coverage track manipulation was performed using the script manipulate_wig (https://github.com/tjparnell/HCI-Scripts).

For H3K4me ChIP-seq in a *set1Δ* background, published data^42^ was re-analyzed. Raw sequence reads for the H3K4me ChIP in WT (BY4741) and *set1Δ* were aligned and processed using our pipeline. Since the chromatin fragments were bar-coded before mixing, immunoprecipitation and sequencing, we utilized the same qChIP-seq analysis methods to calibrate the ChIP sequence depth. The BY4741 reads were treated as the reference genome, while the *set1Δ* reads were treated as the experimental genome.

ChIP-nexus sequence reads were pre-processed before alignment using custom scripts (https://github.com/tjparnell/HCI-Scripts). This removed the random (6 bp) and fixed (4 bp) bar code sequence from the read, storing the random sequence in the read name. After alignment, reads mapping to duplicate coordinates were checked for the random barcode, and duplicates with identical bar codes were discarded, saving the best alignment.

For high level gene analysis from all ChIP experiments, read coverage or fold enrichment was mapped around TSSs using BioToolBox get_relative_data in 20 bp windows from −800 to +800 bp from mapped TSSs, avoiding neighbouring genes, as described^68^. Subsequent analysis and data processing steps were performed using programs available within the BioToolBox package. For histone turnover, normalized-read counts were summed within 20 bp windows flanking the TSS, and a ratio derived between new histone (Flag ChIP) counts and old histone (V5 ChIP) counts. A statistical *P*-value score was generated by a simple parametric repeated-measures ANOVA between corresponding windows of WT and mutant for each gene group (global, common up or down regulated genes, Ribi genes, and so on); *P*-values were converted to *q*-values using R programme. Heat maps were generated with Java Treeview and graphs were generated with GraphPad Prism.

### Data availability

Previously published data from Chabbert *et al*.^42^ were retrieved from ENA accession number ERP007035. Genome-wide Rpb3 and Rpo21 occupancy data from Venters and Pugh^51^ were retrieved from ArrayExpress under accessions numbers E-MEXP-1676 and E-MEXP-1677. Transcription rates for yeast genes from Holstege *et al*.^52^ were obtained from http://www.wi.mit.edu/young/expression.html. RNA-seq, ChIP-seq and MNase-seq data generated in this paper have been deposited at the NCBI Gene Expression Omnibus (GEO) under accession number GSE73407.

## Additional information

**How to cite this article:** Ramakrishnan, S. *et al*. Counteracting H3K4 methylation modulators Set1 and Jhd2 co-regulate chromatin dynamics and gene transcription. *Nat. Commun.* 7:11949 doi: 10.1038/ncomms11949 (2016).

## Supplementary Material

Supplementary Information Supplementary Figures 1-22, Supplementary Tables 1-2.

Supplementary Data 1 Stranded RNA-seq data for sense transcripts upon deletion of *SET1* or *JHD2* or upon overexpression of wild type *SET1* or the hyperactive *SET1G990E* mutant.

Supplementary Data 2 Stranded RNA-seq data for antisense transcripts upon deletion of *SET1* or *JHD2* or upon overexpression of wild type *SET1* or the hyperactive *SET1G990E* mutant.

Supplementary Data 3 Gene ontology (GO) analysis of sense transcripts commonly affected in *set1Δ* or *jhd2Δ* mutant or in all four mutants (*set1Δ*, *jhd2Δ* or in strains overexpressing wild type *SET1* or the *SET1G990E* mutant)

Supplementary Data 4 Transcription rates and GO terms for genes in the k-means clusters co-regulated by Set1 and Jhd2.

## Figures and Tables

**Table 1:** Metrics for sense and antisense transcripts with ≥1.5-fold increase (up) or decrease (down) in expression (FDR ≤5%) in *jhd2Δ* or *set1Δ* relative to WT.

	Sense			Antisense		
	Total	Up	Down	Total	Up	Down
*jhd2Δ*	655	387	268	114	56	58
*set1Δ*	463	321	142	268	155	113

**Table 2:** Total number of sense or antisense transcripts undergoing an opposite change in expression (FDR ≤5%) in *jhd2Δ* and *set1Δ* mutants.

	Down regulated in *jhd2Δ*		Down regulated in *set1Δ*	
	Sense	Antisense	Sense	Antisense
Up regulated in *jhd2Δ*	−	−	0	1
Up regulated in *set1Δ*	13	2	−	−

**Table 3:** Chart shows transcription rates for genes within the six *k*-means clusters.

Cluster	mRNA/h	*P*-value	GO terms
1	4.0–15.9	0.001	Ribosomal large subunit biogenesis
2	1.0–3.9	<0.001	Metabolic process, RNA metabolic process, Mitotic cell cycle
2	4.0–15.9	0.02	Ribosomal biogenesis, Metabolic process, ATP metabolic proces
3	<1.0	0.002	
5	1.0–3.9	0.029	Transcription, response to stimulus, transport, chromatin modification, cell cycle
5	4.0–15.9	<0.001	Proteolysis, alpha-amino acid metabolic process
5	>50.0	0.004	Translation
6	4.0–15.9	<0.001	Transport, oxoacid metabolic process
6	16.0–50.0	<0.001	Small molecule metabolic process, steroid biosynthetic process
6	>50.0	<0.001	Translation, glycolytic process

They were determined by intersecting genes in each cluster with groups of yeast genes classified based on increasing transcription rates (mRNA/h)^52^: <1, 1–3.9, 4–15.9, 16–50 and >50. Correlation *P*-value for each intersection is shown. Gene ontology (GO) terms enriched amongst the common intersecting genes are also listed.
