# Supplementary material for: Counteracting H3K4 methylation modulators Set1 and Jhd2 co-regulate chromatin dynamics and gene transcription
Source: Nat Commun. 2016 Jun 21;7:11949. doi: 10.1038/ncomms11949 (PMC4919544; doi:10.1038/ncomms11949)
Supplement: Supplementary Information — Supplementary Figures 1-22, Supplementary Tables 1-2. [file ncomms11949-s1.pdf]

Strand	Gene name	Fold change *jhd2Δ* vs *WT*	Fold change *set1Δ* vs *WT*
s	***YMR118C***	0.60 ↓	1.59 ↑
s	***MTD1***	0.58 ↓	1.57 ↑
s	***SOM1***	0.65 ↓	1.53 ↑
s	***ARG1***	0.58 ↓	1.52 ↑
s	***YJR079W***	0.65 ↓	1.50 ↑
s	***BNA2***	0.66 ↓	1.90 ↑
s	***YJR154W***	0.65 ↓	1.75 ↑
s	***DSF1***	0.50 ↓	1.58 ↑
s	***YGL138C***	0.60 ↓	1.60 ↑
s	***GON3***	0.49 ↓	1.56 ↑
s	***FLO1***	0.58 ↓	5.88 ↑
s	***SPO75***	0.59 ↓	2.75 ↑
s	***YSN1***	0.62 ↓	1.71 ↑
as	***FLO10***	0.55 ↓	2.12 ↑
as	***SKN1***	0.55 ↓	1.85 ↑
as	***YDR061W***	1.68 ↑	0.61 ↓

Supplementary Figure 1. List of sense and antisense transcripts counter-regulated by Set1 and Jhd2. Fold change in sense (s) or antisense (as) transcript levels for the indicated genes in *jhd2Δ* or *set1Δ* mutant relative to the control wild type (WT) are shown. Fold change in transcript levels were calculated using sequencing reads obtained from three independent RNA samples each isolated for control WT and deletion mutants (*jhd2Δ* or *set1Δ*). Red arrows denote down-regulation of the transcript in the mutant, and green arrows indicate up-regulation of the transcript in the mutant.

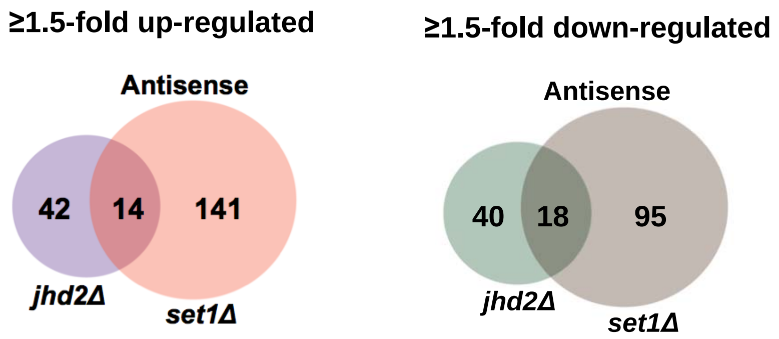

Supplementary Figure 2. Venn diagrams showing the number of up- or down-regulated antisense transcripts unique to and common to *jhd2Δ* and *set1Δ* mutants. Intersection *p-value* $<10^{-4}$ (hypergeometric test).

a

	jhd2Δ	*set1Δ*
SER3	10.97	7.52
CHA1	1.49	2.38

Fold down-regulated relative to wild-type

b

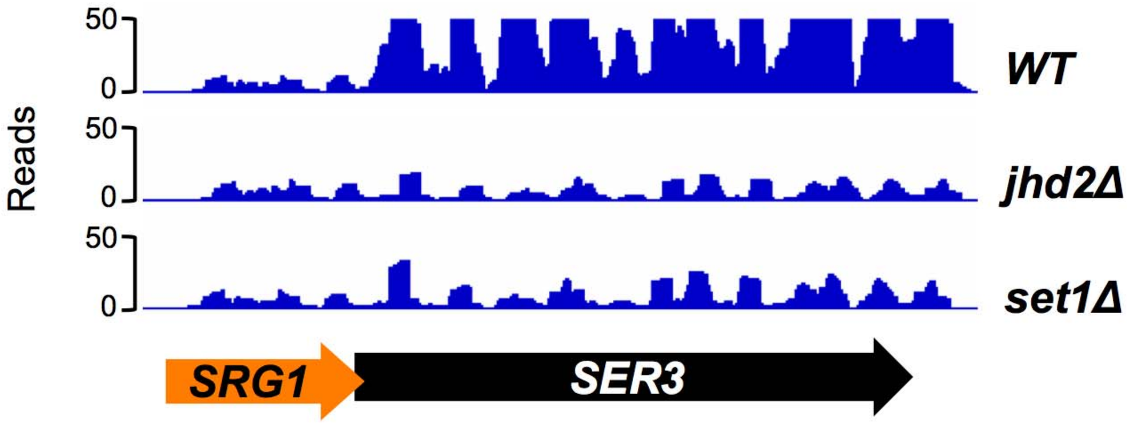

Supplementary Figure 3. *SER3* and *CHA1* genes involved in serine metabolism are target genes of Set1 and Jhd2. (a) Fold decrease in the RNA-seq reads for *SER3* and *CHA1* transcripts in *jhd2Δ* or *set1Δ* mutant relative to control wild type. **(b)** *SRG1* transcription is unaffected by the absence of Set1 or Jhd2. RNA-seq reads obtained for *SRG1* and *SER3* transcripts in control wild type (WT), *jhd2Δ* and *set1Δ* are shown. *SRG1* codes for an upstream non-coding RNA involved in negatively regulating *SER3* transcription.

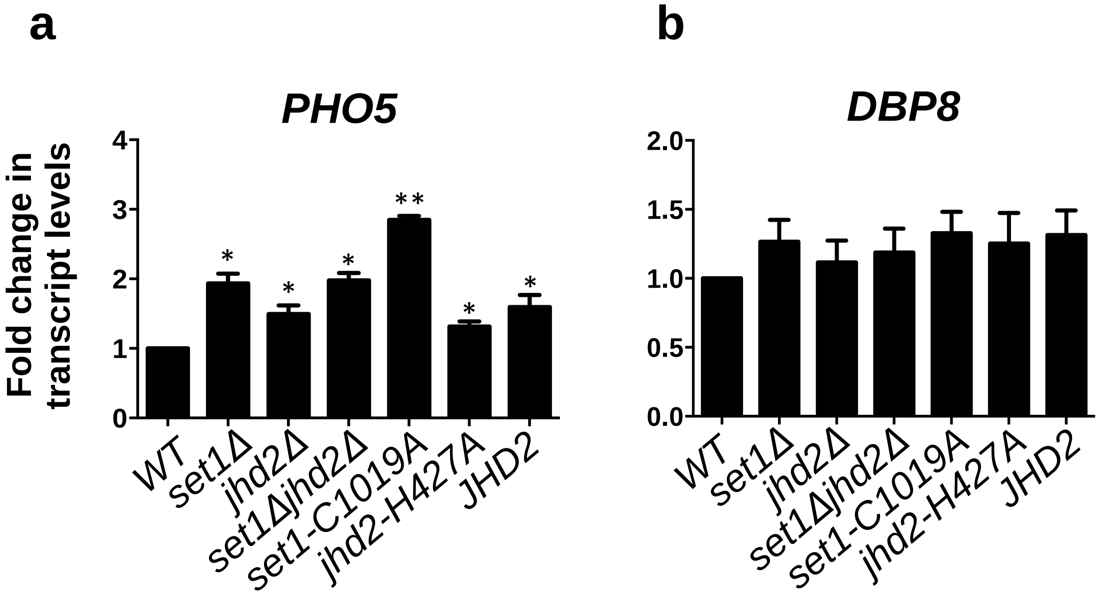

Supplementary Figure 4. Set1 and Jhd2 control transcriptional repression or attenuation at *PHO5* and *DBP8* genes. Fold-change in *PHO5* and *DBP8* sense transcript levels in deletion mutants (*set1Δ, jhd2Δ* and *set1Δjhd2Δ*), catalytic-dead mutants (*set1-C1019A* and *jhd2-H427A*) or a strain overexpressing Jhd2 (*JHD2*) relative to the control WT strain are shown. Error bars denote ±SEM from four independent experiments (n=4). Statistical significance calculated using Student's t-test, **p-value* ≤0.05, ***p-value* $<10^{-4}$.

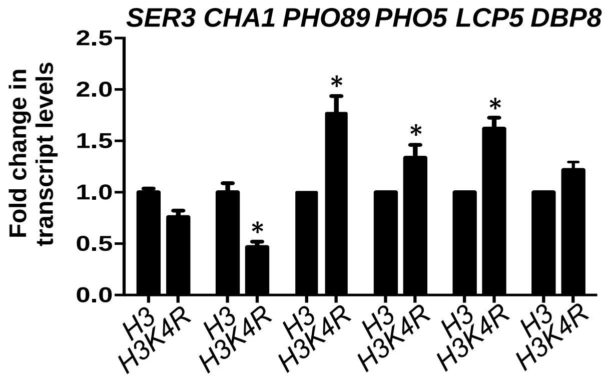

Supplementary Figure 5. Histone H3K4R mutation alters target gene expression. Fold-change in transcript levels for the indicated target genes in the *H3K4R* mutant relative to wild type H3 strain are shown. Error bars denote ±SEM from four independent experiments (n=4). Statistical significance calculated using Student's t-test, **p-value* <0.05.

a

1.5-fold change FDR ≤5%

	SENSE			ANTISENSE		
	**Total**	**Up**	**Down**	**Total**	**Up**	**Down**
jhd2Δ	**655**	387	268	**114**	56	58
set1Δ	**463**	321	142	**268**	155	113
FlagSET1	**403**	206	197	**83**	30	53
FlagSET1-G990E	**630**	363	267	**146**	71	75

2-fold change FDR ≤1%

	SENSE			ANTISENSE		
	**Total**	**Up**	**Down**	**Total**	**Up**	**Down**
jhd2Δ	**149**	101	48	**26**	13	13
set1Δ	**71**	53	18	**99**	61	38
FlagSET1	**93**	36	57	**14**	4	10
FlagSET1-G990E	**162**	92	70	**40**	25	15

b

Antisense transcripts up-regulated ≥1.5-fold relative to wild-type

Antisense transcripts down-regulated ≥1.5-fold relative to wild-type

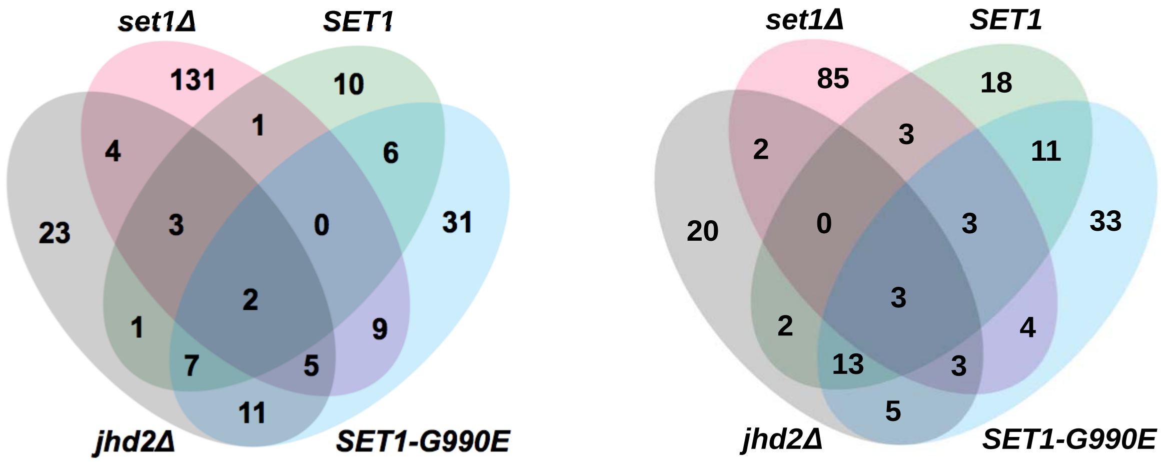

Supplementary Figure 6. Sense and antisense transcription are altered upon deletion of *SET1* or *JHD2* or following overexpression of *SET1* or hyperactive *SET1-G990E*. (a) The total number of sense or antisense transcripts undergoing a 1.5-fold (at a false discovery rate (FDR) ≤5%) or a 2-fold (FDR ≤1%) change in expression in the four indicated mutants compared to the control wild type are shown. The numbers of sense or antisense transcripts either up- or down-regulated in each of the four mutant strains are also indicated. **(b)** Four-way Venn diagrams showing the number of unique or shared up- or down-regulated antisense transcripts in each combination of the indicated deletion or overexpression mutant strains. Intersection *p-value* $<10^{-4}$ (hypergeometric test).

a

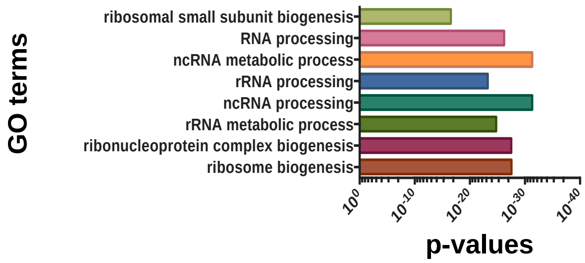

b

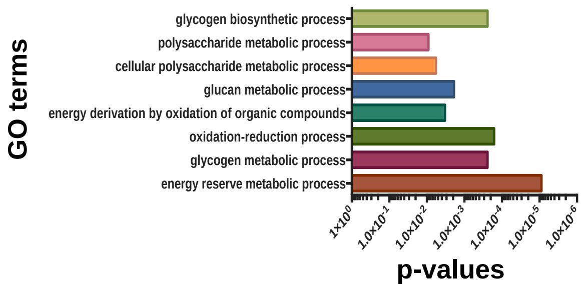

Supplementary Figure 7. Genes for glycogen metabolism and ribosome biogenesis are sensitive to the deletion of *SET1* or *JHD2* or overexpression of *SET1* or hyperactive *SET1-G990E*. (a) GO terms significantly enriched in the up-regulated sense transcripts common to deletion (*set1Δ* or *jhd2Δ*) and overexpression mutants (*SET1* or *SET1-G990E*). **(b)** GO terms enriched in sense transcripts down-regulated in all four mutant strains.

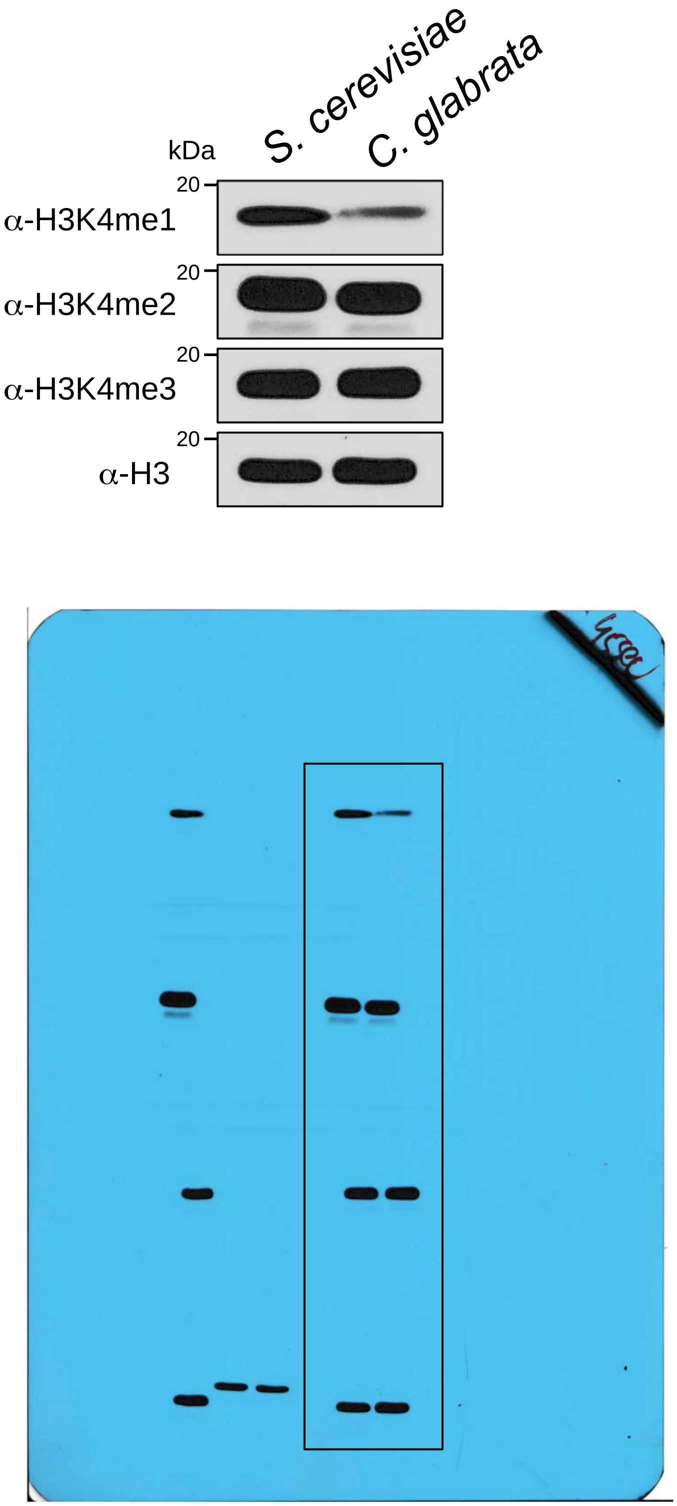

Supplementary Figure 8. H3K4 methylation occurs in *Candida glabrata*. Western blots showing H3K4me1, H3K4me2 and H3K4me3 levels in whole cell lysates prepared from *S. cerevisiae* (experimental genome) and *C. glabrata* (reference genome). H3 serves as loading control. The full-image of the blot is also shown and panels at the top are indicated.

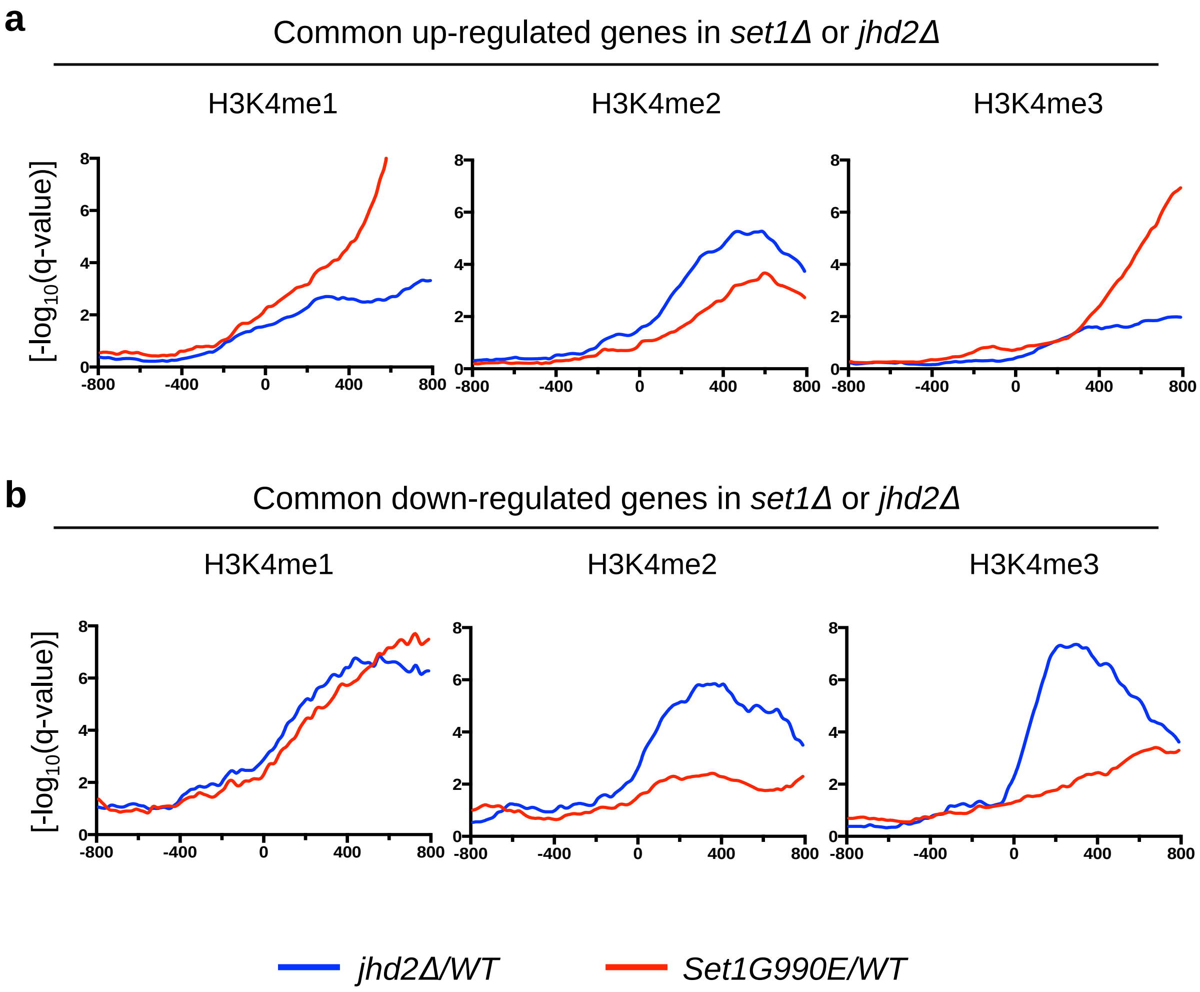

Supplementary Figure 9. Statistical significance are shown for the changes in H3K4 methylation levels at Set1 and Jhd2 co-repressed (**a**) or co-activated (**b**) target genes in *jhd2Δ* or *Set1G990E* mutant compared to wild type.

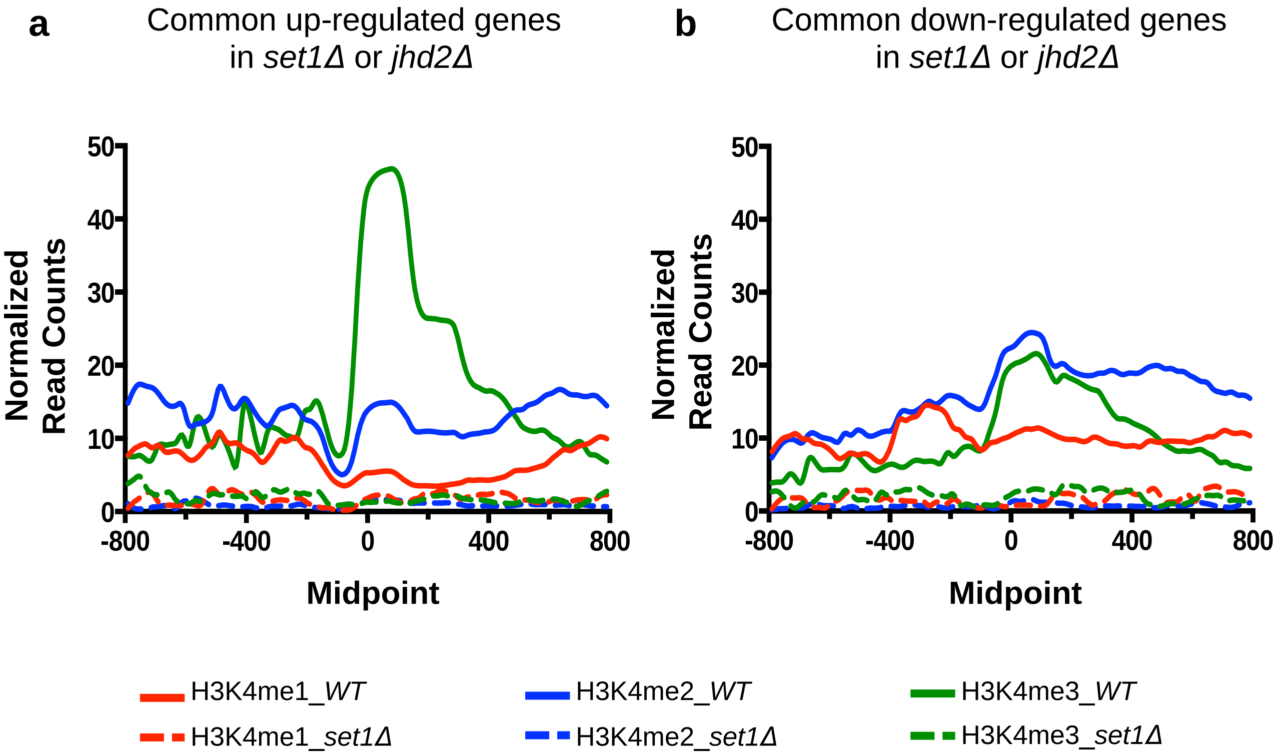

Supplementary Figure 10. H3K4 methylation is abolished at shared target genes in the *set1Δ* mutant. Normalized read counts profiles for H3K4 monomethyl (me1), dimethyl (me2) and trimethy (me3) marks across 800 bp region upstream (-) or downstream (+) of the TSSs (0) at genes co-repressed (**a**) or co-activated (**b**) by Set1 and Jhd2 in BY4741 (WT) and *set1Δ* null mutant. Read counts for *set1Δ* null mutant and its control wild type strain BY4741 were obtained from published datasets of Chabbert et al (2015).

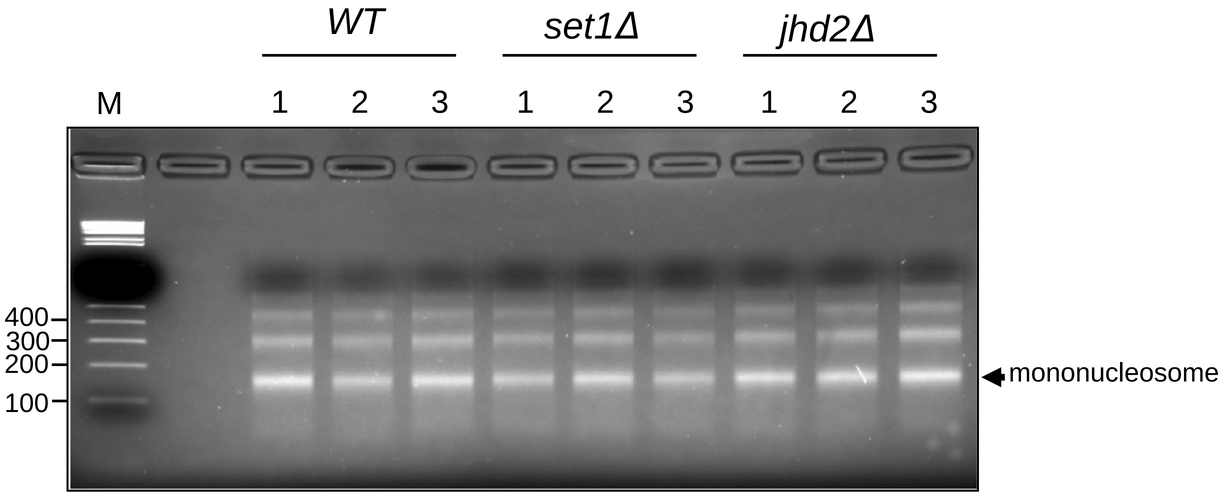

Supplementary Figure 11. Bulk chromatin structure in wild type, *set1Δ* and *jhd2Δ* strains. A 2% agarose gel stained with ethidium bromide and containing limited micrococcal nuclease (MNase) digested DNA isolated from three independent cultures each for the wild type (WT), *set1Δ* and *jhd2Δ* strains is shown. These MNase-digested DNA were subjected to library preparation. Adaptor-ligated DNA corresponding to mononucleosomes was size selected by gel purification and subjected to next generation sequencing. M, 1kb DNA ladder.

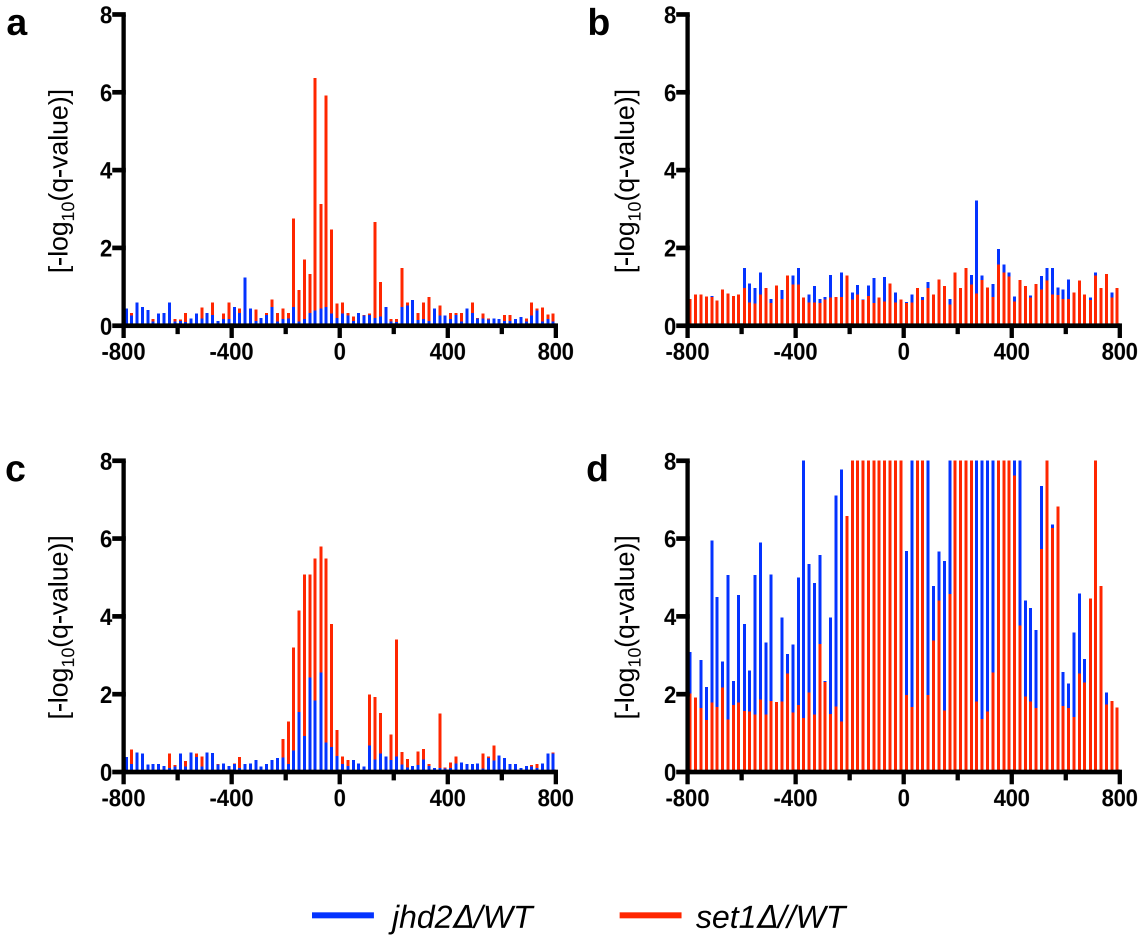

Supplementary Figure 12. Statistical significance are shown for the histone turnover changes at Set1 and Jhd2 co-repressed (**a**) or co-activated (**b**) target genes, all yeast ribi genes (**c**) and across all yeast genes (**d**) in *jhd2Δ* or *set1Δ* mutant compared to wild type.

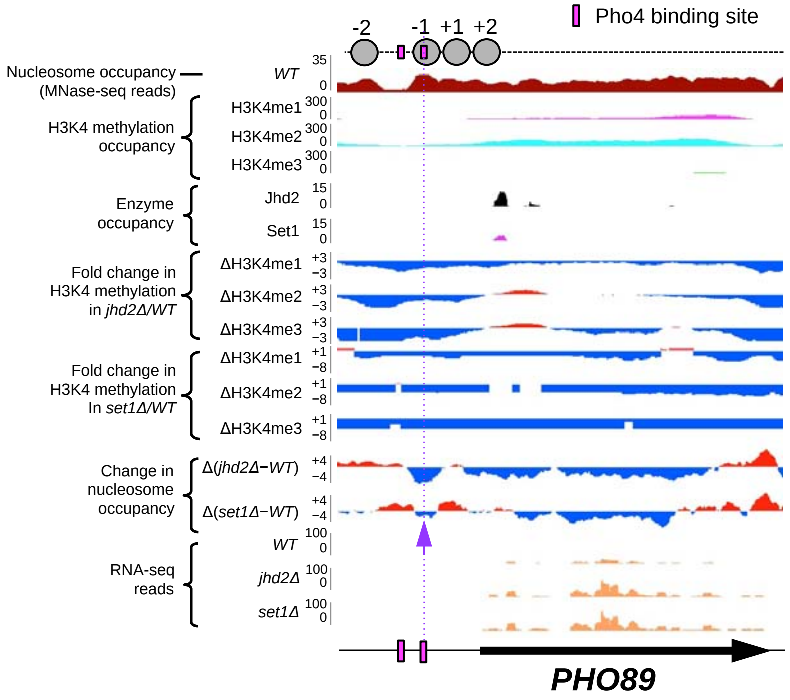

Supplementary Figure 13. Occupancy of a *PHO89* promoter nucleosome containing the Pho4 binding site is reduced in *set1Δ* or *jhd2Δ* mutant. Changes to H3K4 methylation levels, nucleosomal occupancy and transcripts at the *PHO89* gene in *jhd2Δ* or *set1Δ* are shown. The normal nucleosomal, H3K4 methylation, Jhd2 and Set1 occupancies at these genes are also shown: Reads for mononucleosomal DNA in wild type (WT) shows the native chromatin organization (*brown*). Reads for H3K4 monomethyl (me1, *magenta*), H3K4 dimethyl (me2, *cyan*) and H3K4 trimethyl (me3, *green*) marks in WT shows their distribution over the *PHO89* gene. Jhd2 (*black*) and Set1 (*pink*) occupancy (qpois) in WT strain at the *PHO89* gene are shown. Fold change in the levels of H3K4me1, H3K4me2 and H3K4me3 marks in *jhd2Δ* or *set1Δ* relative to that in WT are shown. Fold change in H3K4 methylation in *set1Δ* relative to WT was calculated using data from Chabbert et al (2015). Increase or decrease in a given H3K4 methyl mark in the mutant is shown in red or blue, respectively. Mononucleosomal DNA from WT were subtracted from those obtained from *jhd2Δ* or *set1Δ* and visualized using a genome browser. Gain or loss in nucleosome occupancy in the mutant is represented in red or blue, respectively. RNA-seq reads for *PHO89* transcript levels in *WT*, *jhd2Δ* or *set1Δ* strains are also shown (*orange*). Schematic diagram at top shows the nucleosome organization over promoter and TSS regions of the *PHO89* gene. *Circles*, well-positioned promoter nucleosomes; *black arrow*, TSS; and *pink box*, Pho4 binding sites, predicted from the published studies of Badis et al. (2008) and Harbison et al. (2004). *Grape colored arrow and dotted line* indicate decrease in nucleosomal occupancy at one of the two predicted Pho4 binding sites.

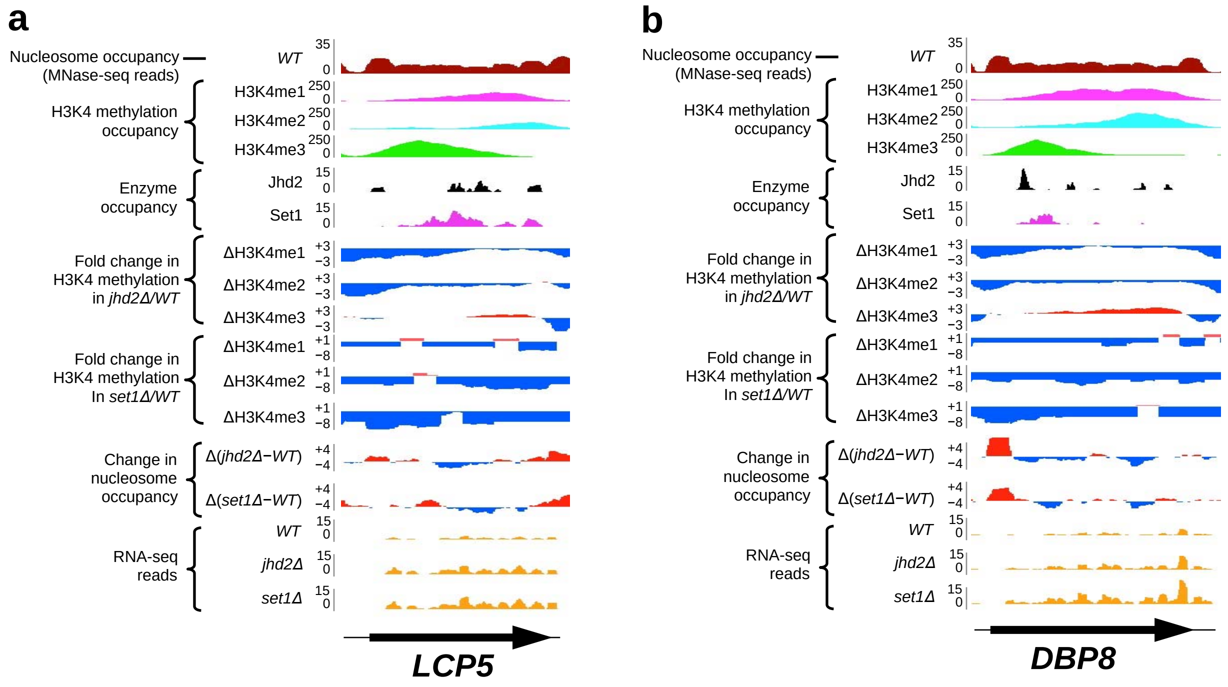

Supplementary Figure 14. Set1 and Jhd2 co-regulate H3K4 methylation, nucleosomal occupancy and expression of candidate Ribi genes, *LCP5* and *DBP8*. (a-b) Changes in H3K4 methylation levels, nucleosomal occupancy and transcript levels for *LCP5* and *DBP8* genes in *jhd2Δ* or *set1Δ* are shown. The normal nucleosomal, H3K4 methylation, Jhd2 and Set1 occupancies at these genes are also shown. Reads for mononucleosomal DNA in wild type (WT) shows the native chromatin organization (*brown*). Reads for H3K4 monomethyl (me1, *magenta*), H3K4 dimethyl (me2, *cyan*) and H3K4 trimethyl (me3, *green*) marks in WT shows their distribution over the *LCP5* and *DBP8* genes. Jhd2 (*black*) and Set1 (*pink*) occupancy (qpois) in WT strain at the *LCP5* and *DBP8* genes are shown. Fold change in the levels of H3K4me1, H3K4me2 and H3K4me3 marks in *jhd2Δ* or *set1Δ* relative to that in WT are shown. Fold change in H3K4 methylation in *set1Δ* relative to WT was calculated using data from Chabbert et al (2015). Increase or decrease in a given H3K4 methyl mark in the mutant is shown in red or blue, respectively. Mononucleosomal DNA from WT were subtracted from those obtained from *jhd2Δ* or *set1Δ* and visualized using a genome browser. Gain or loss in nucleosome occupancy in the mutant is represented in red or blue, respectively. RNA-seq reads for *LCP5* and *DBP8* transcript levels in *WT*, *jhd2Δ* or *set1Δ* strains are also shown (*orange*).

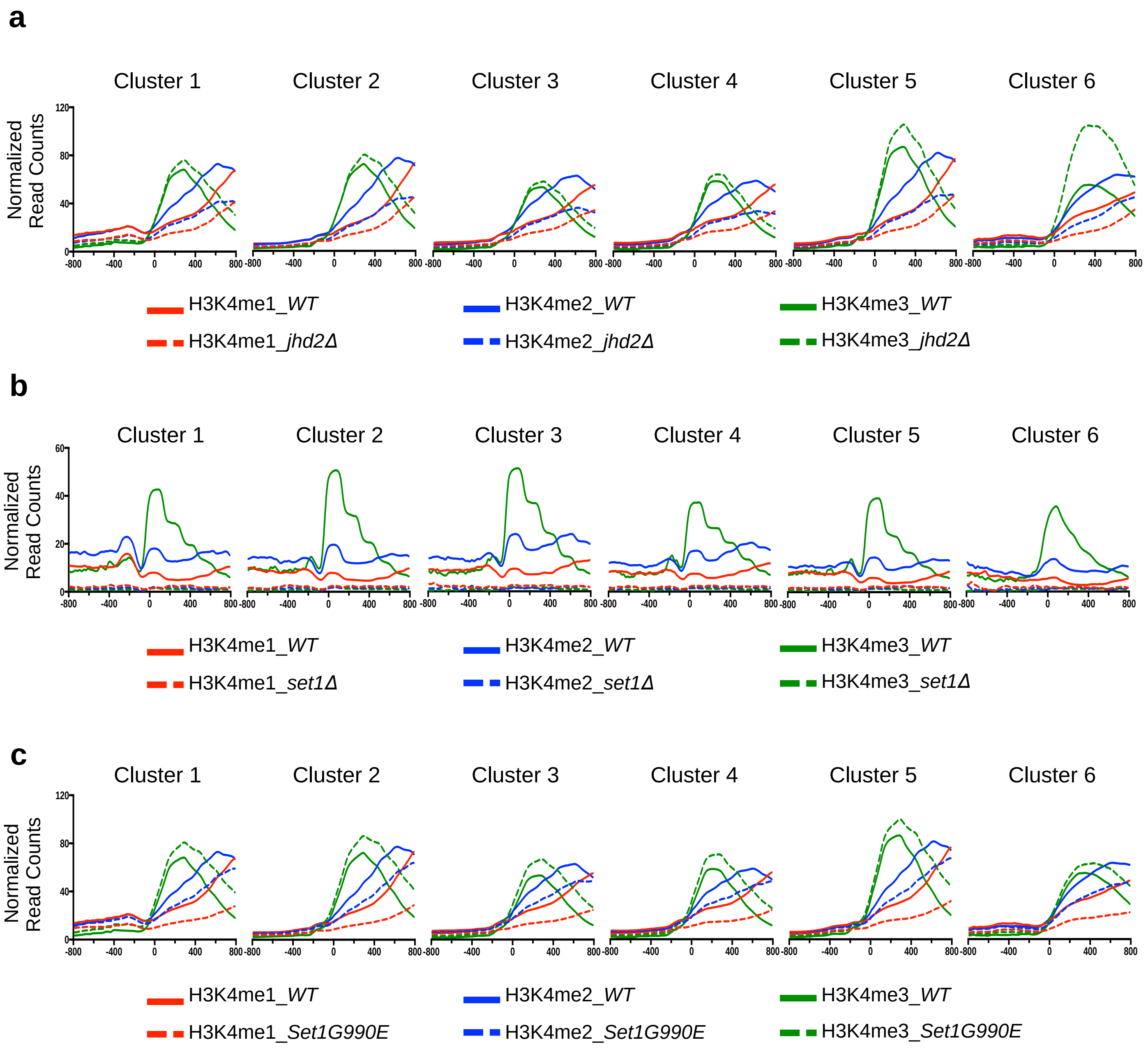

Supplementary Figure 15. Deletion of *JHD2* or *SET1* or the expression of hyperactive Set1-G990E alters H3K4 methylation levels at all six k-means clusters. Normalized read counts profiles for H3K4me1, H3K4me2 and H3K4me3 marks across 800 bp region upstream (-) or downstream (+) of the TSSs (0) at genes within the six k-means clusters (Fig. 6a-b) in control wild type (WT), *jhd2Δ* or *set1Δ* null mutant and hyperactive Set1-G990E expressing strain are shown. Read counts for *set1Δ* null mutant and its control wild type strain BY4741were obtained from published datasets of Chabbert et al (2015).

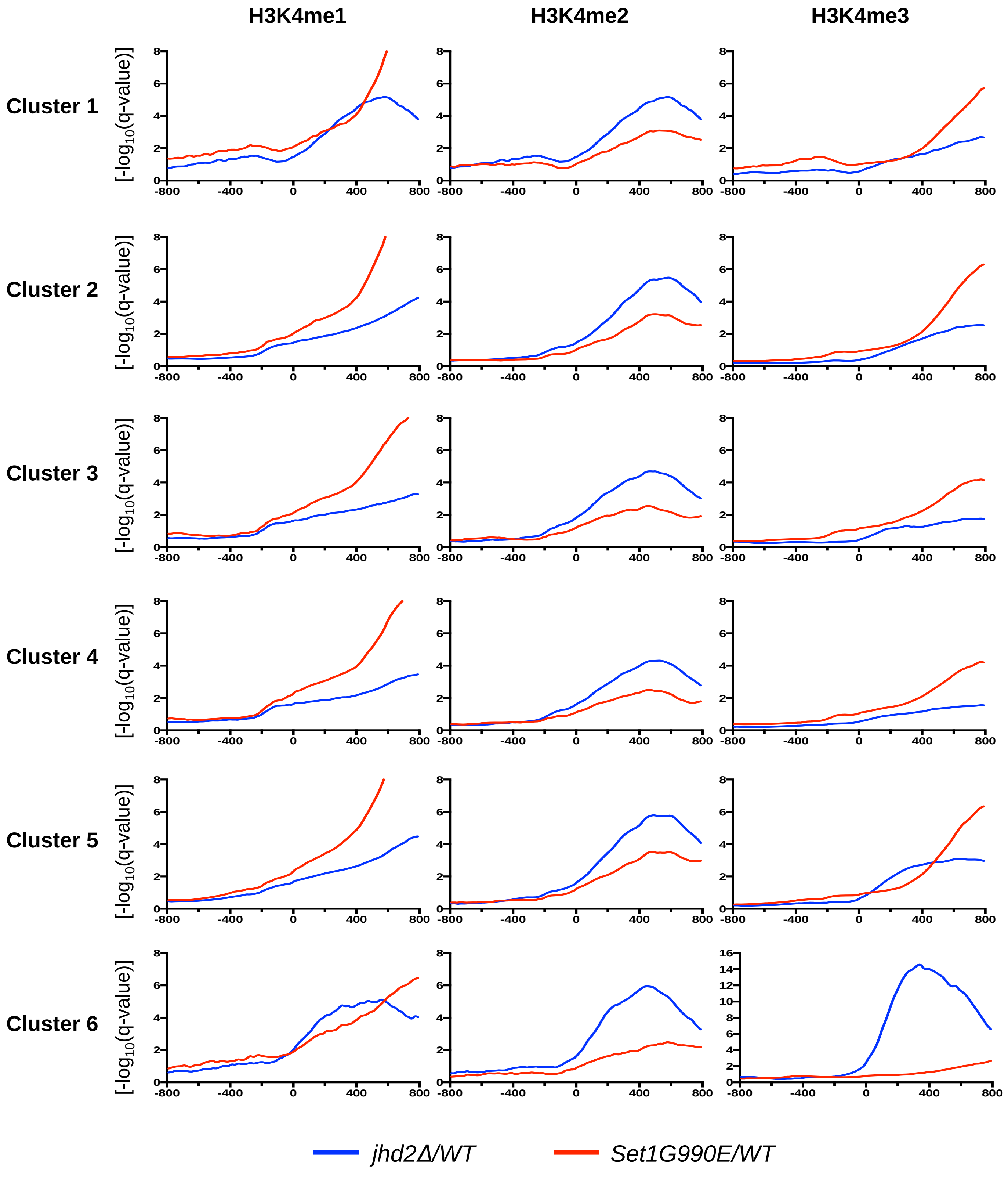

Supplementary Figure 16. Statistical significance are shown for the changes in H3K4 methylation levels at the six gene clusters in *jhd2Δ* or *Set1G990E* mutant compared to control wild type.

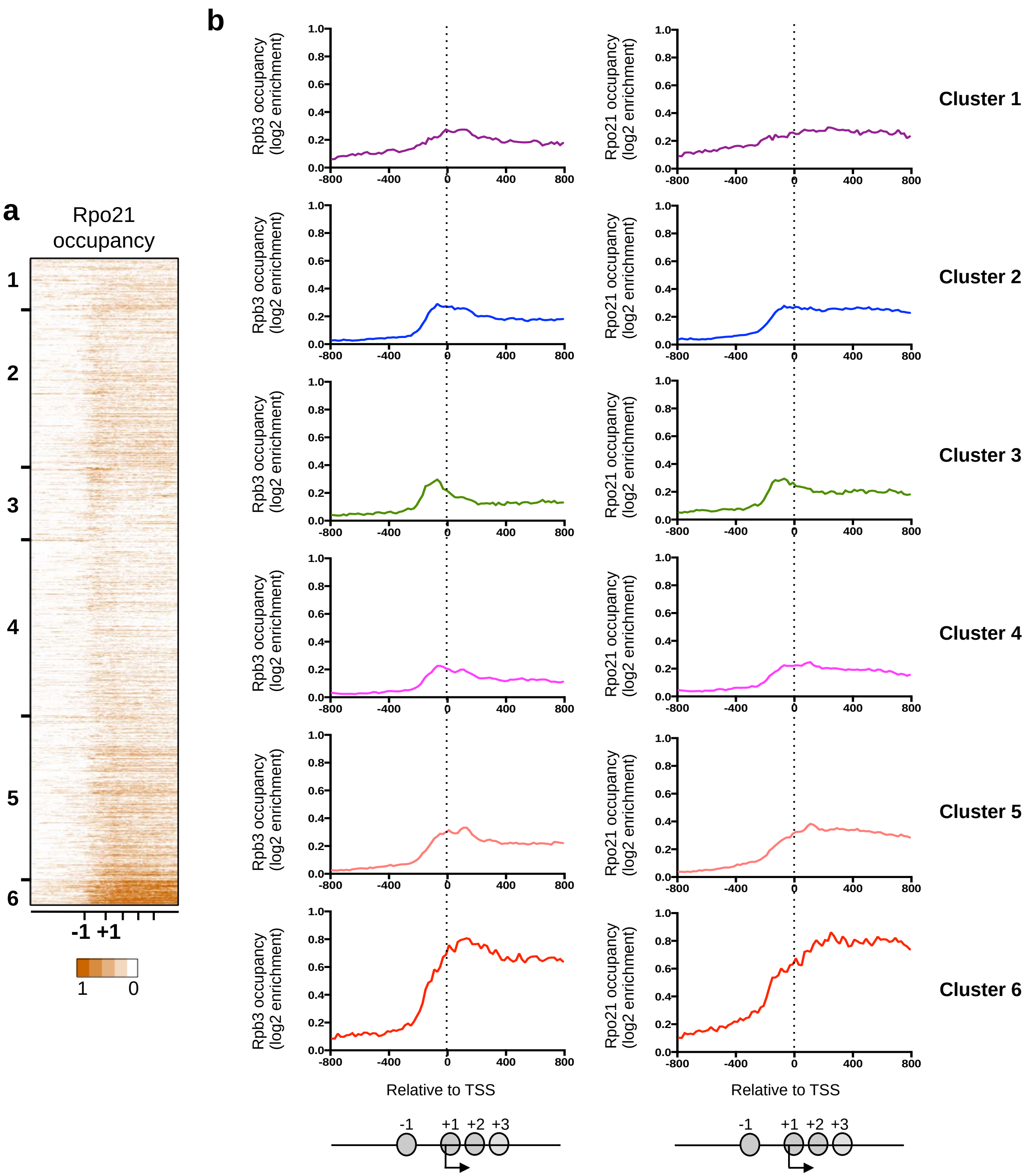

Supplementary Figure 17. Occupancy and distribution of Rpb3 and Rpo21 at all six k-means clusters. (a) Heat map for Rpo21 occupancy within the six k-means clusters, using data from Venters and Pugh (2009), is shown. Rpo21 occupancy above global mean is in orange. The mean occupancy profiles over each of the six k-means clusters (shown in Fig. 6a-b) are shown for two RNA polymerase II (Pol2) subunits, Rpb3 and Rpo21 (Venters and Pugh, 2009). Dotted line marks TSS (0). In the schematic at the bottom, circles denote the positions of -1, +1 and coding region nucleosomes and the arrow denotes the TSS.

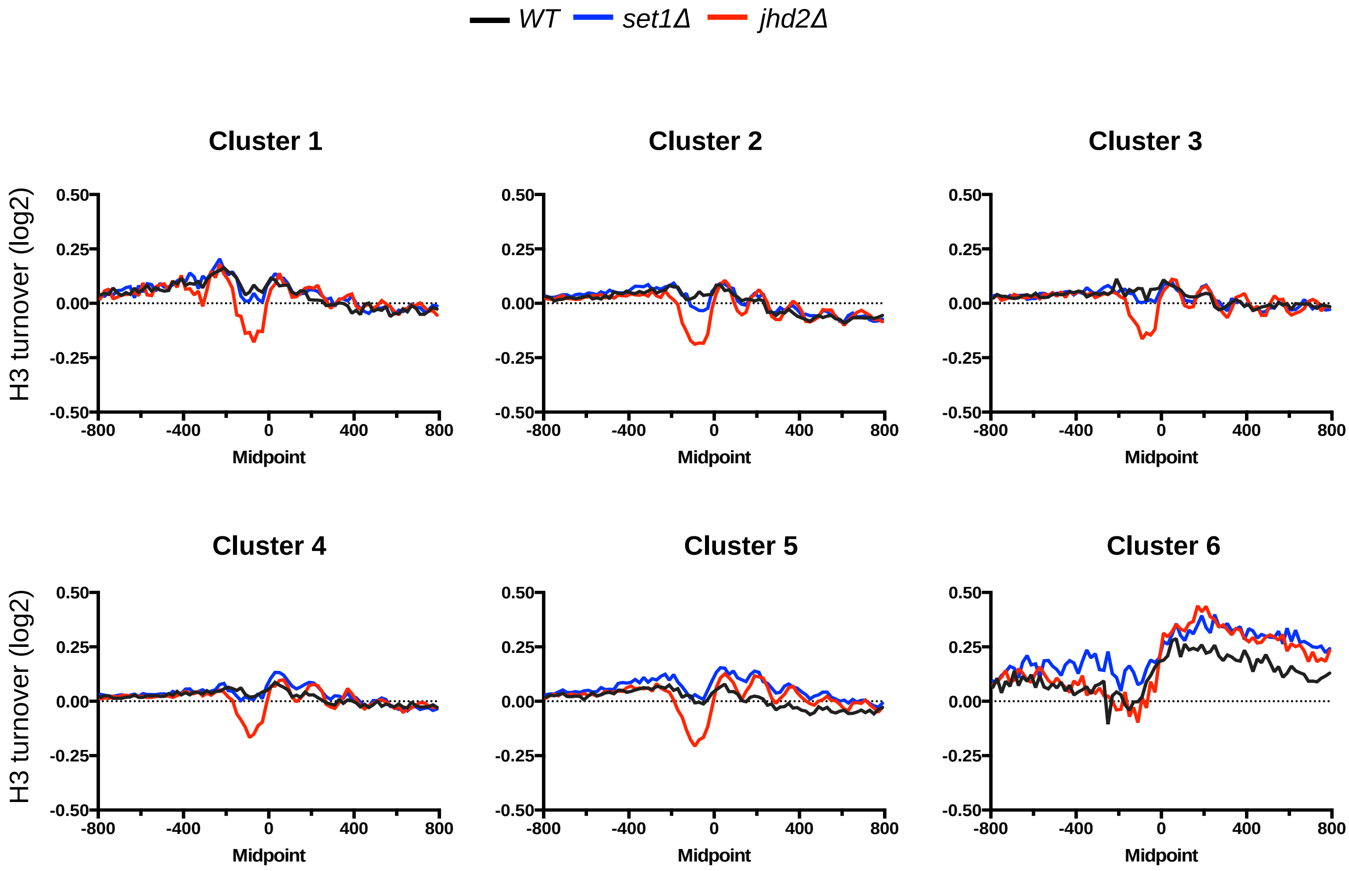

Supplementary Figure 18. Set1 and/or Jhd2 controls nucleosomal turnover at genes within the six k-means clusters. Mean profile for histone H3 turnover across 800 bp region upstream (-) or downstream (+) of the TSSs (0) at genes within the six k-means clusters (Fig. 6a-b) are shown in wild type (WT), *set1Δ* and *jhd2Δ* strains.

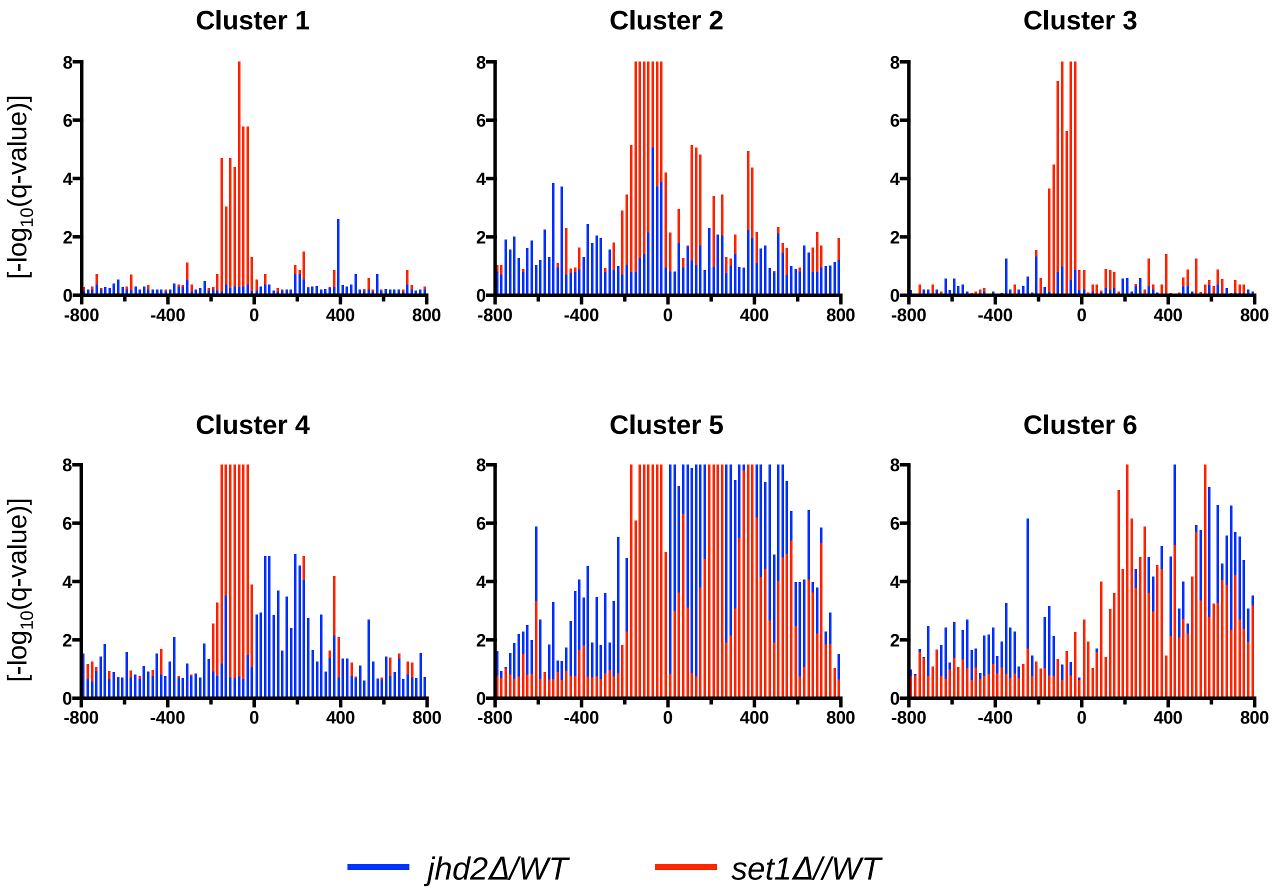

Supplementary Figure 19. Statistical significance are shown for the histone turnover changes at the six k-means clusters in *jhd2Δ* or *set1Δ* mutant compared to wild type.

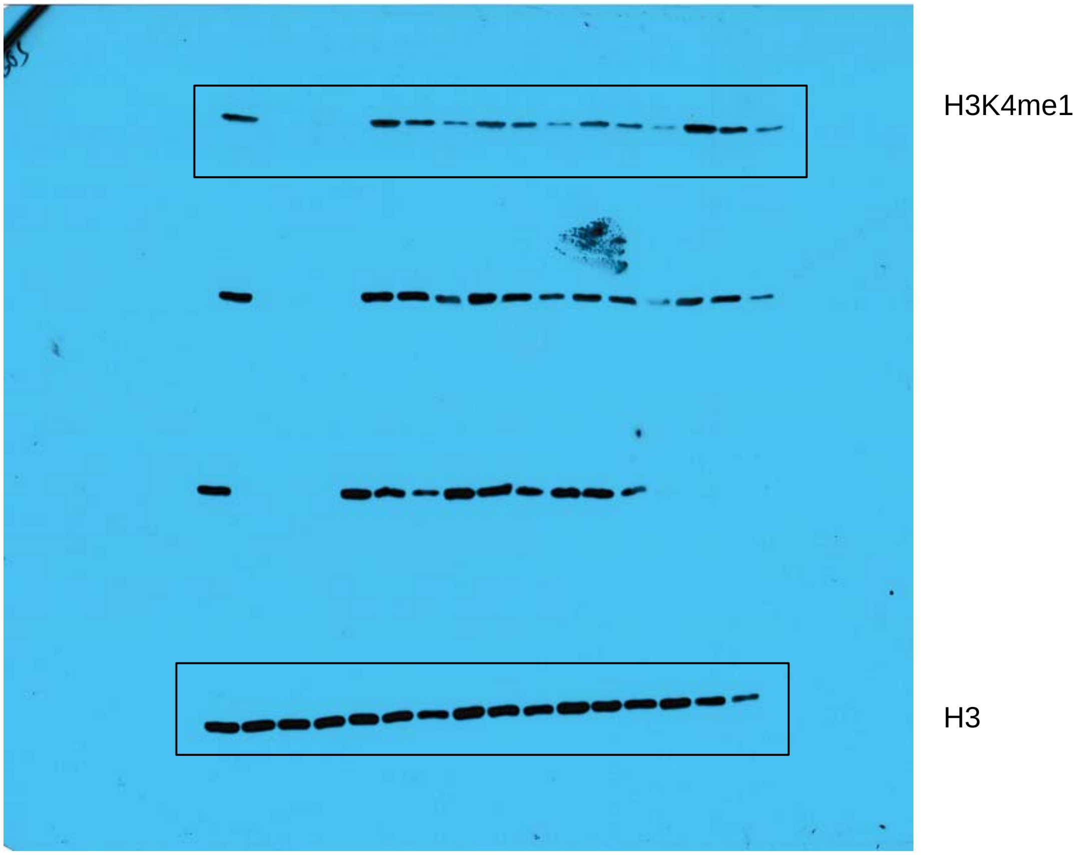

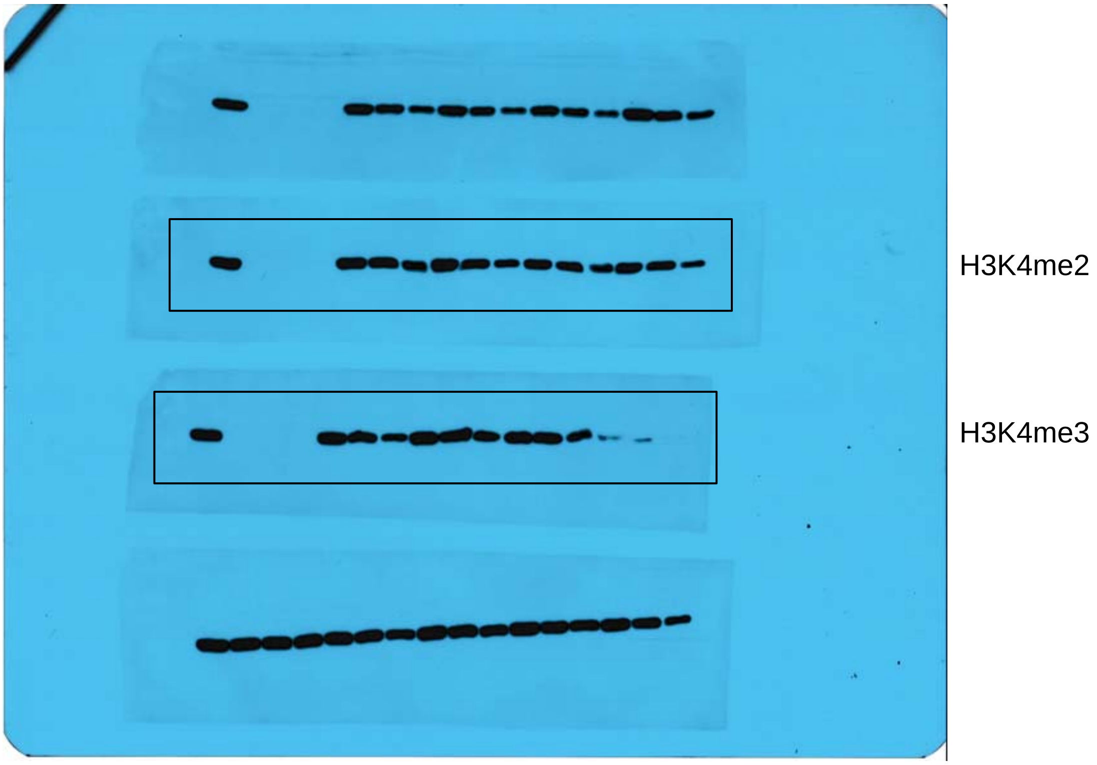

Supplementary Figure 20. Full-length image of the blots shown in Fig. 2a. Panels displayed in Fig. 2a are shown within rectangles.

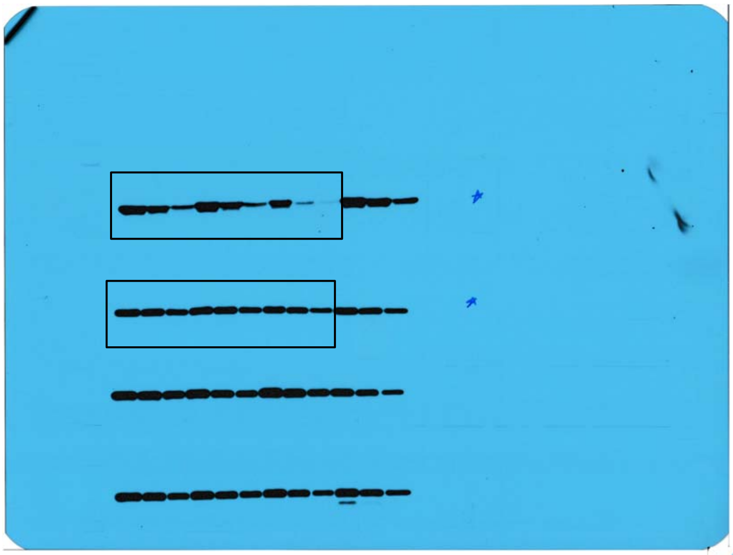

H3K4me1 H3K4me2

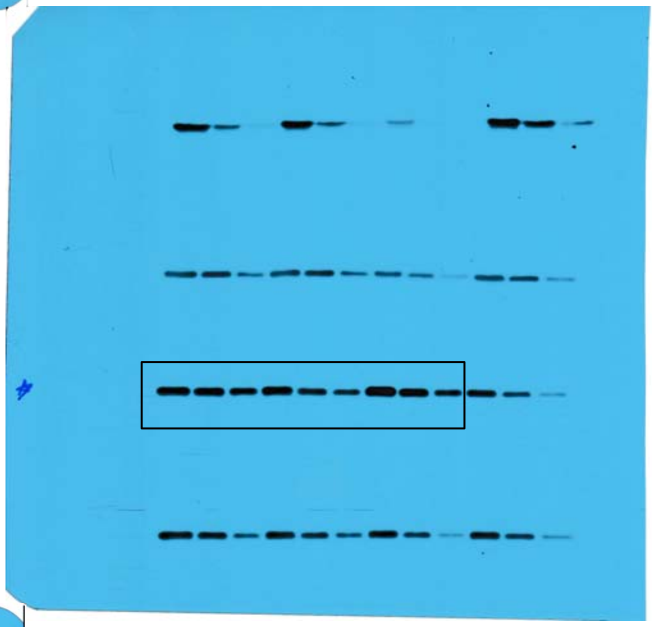

H3K4me3

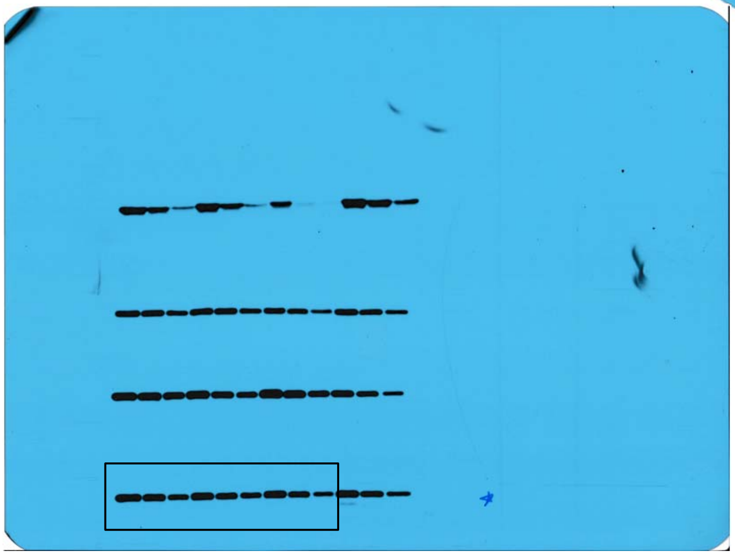

H3

Supplementary Figure 21. Full-length image of the blots shown in Fig. 2d. Panels displayed in Fig. 2d are shown within rectangles.

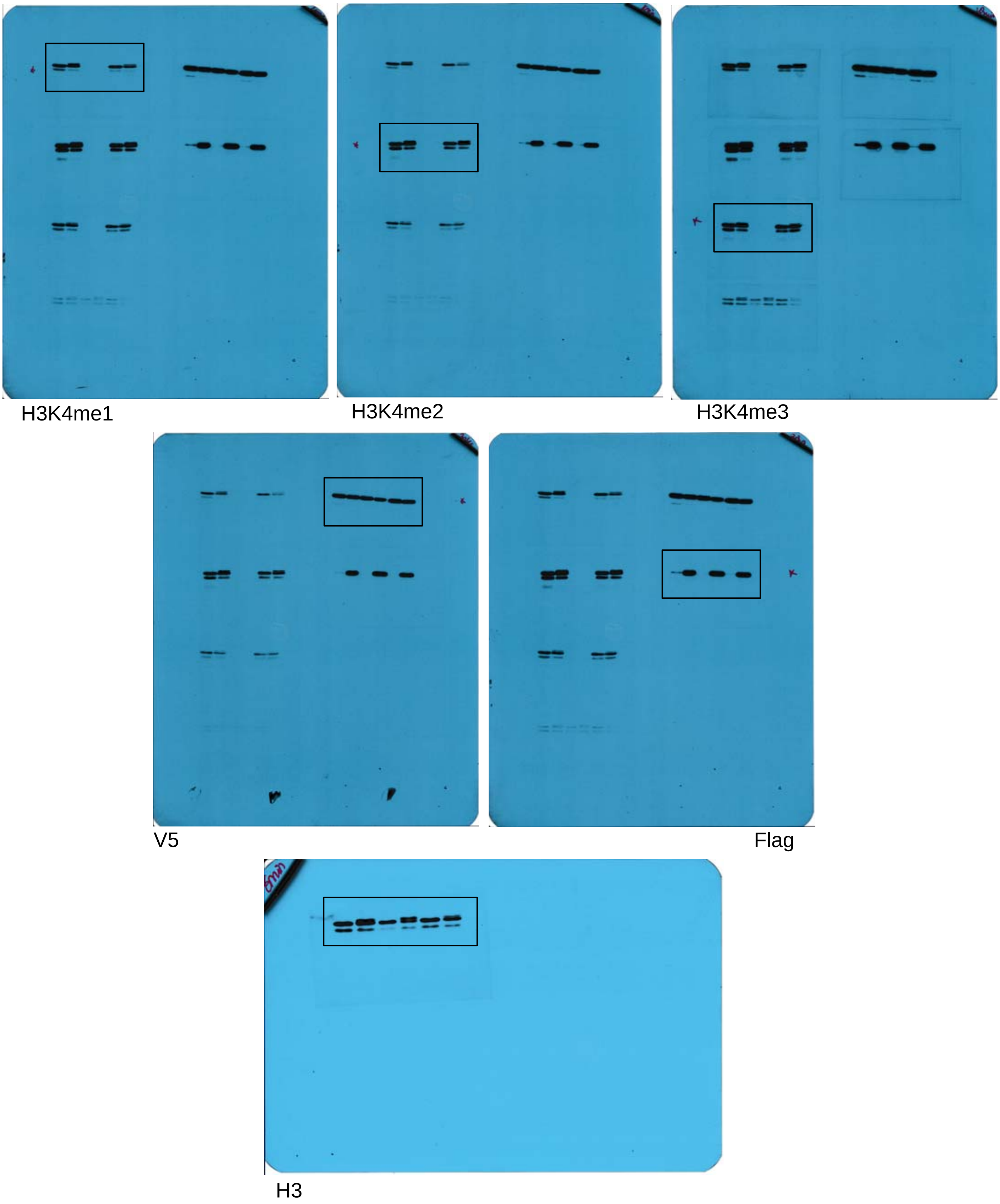

Supplementary Figure 22. Full-length image of the blots shown in in Fig. 4c. Panels shown are within rectangles.

Supplementary Table 1. ***Saccharomyces cerevisiae*** **strains used**

Strain	Genotype	Source
FY406	*Mat a hta1-htb1Δ hta2-htb2Δ lys2-128δhis3Δ200 ura3-52 pSAB6 [HTA1-HTB1 (CEN URA3*)]	(1)
HTABWT	*HTA1-HTB1 (CEN HIS3*); derived from FY406	(2)
YZS515	*Mat a ura3-52 leu2-3,112 his3 trp1Δ set1Δ::KanMX6*	(3)
YZS652	*jhd2Δ::KanMX6*; derived from FY406	(2)
YMC42	*ADH1p-FlagSET1::URA3 HTA1-HTB1 (CEN HIS3*); derived from HTABWT	This study
YMC43	*ADH1p-FlagSET1G990E::URA3 HTA1-HTB1 (CEN HIS3*); derived from HTABWT	This study
YMC44	*ADH1p-JHD2-9myc::URA3 HTA1-HTB1 (CEN HIS3*); derived from HTABWT	This study
YMC64	*set1Δ::KanMX6 HTA1-HTB1 (CEN HIS3*); derived from HTABWT	(2)
YMC65	*jhd2Δ::NatMX6 set1Δ::KanMX6 HTA1-HTB1 (CEN HIS3*); derived from YMC64	This study
YMC70	*jhd2Δ::KanMX6 HTA1-HTB1 (CEN HIS3*); derived from YZS652	(2)
YMC78	*ADH1p-jhd2H427A-9myc::URA3 HTA1-HTB1 (CEN HIS3*); derived from YMC70	This study
YMC84	*Jhd2-12V5::KanMX6*; derived from FY406	(2)
YMC85	*Jhd2-12V5::KanMX6 HTA1-HTB1 (CEN HIS3);* derived from YMC84	(2)
YMC127	*8V5-Set1::URA3;* derived from HTABWT	This study
YMC120	P_{TDH3}*-Cre-EBD78::URA3;* derived from HTABWT	This study
YMC122	*HHT1-V5-LoxP-HphMX-LoxP-2FLAG*; derived from YMC120	This study
YMC124	*(hht2-hhf2)Δ::NatMX6*; derived from YMC122	This study
YMC125	*set1Δ::KanMX6*; derived from YMC124	This study
YMC128	*jhd2Δ::KanMX6*; derived from YMC124	This study

1. Hirschhorn, J. N., Bortvin, A. L., Ricupero-Hovasse, S. L., and Winston, F. (1995) A new class of histone H2A mutations in Saccharomyces cerevisiae causes specific transcriptional defects in vivo. *Mol Cell Biol* **15**, 1999-2009
2. Huang, F., Ramakrishnan, S., Pokhrel, S., Pflueger, C., Parnell, T. J., Kasten, M. M., Currie, S. L., Bhachech, N., Horikoshi, M., Graves, B. J., Cairns, B. R., Bhaskara, S., and Chandrasekharan, M. B. (2015) Interaction of the Jhd2 H3K4 demethylase with chromatin is controlled by histone H2A surfaces and restricted by H2B ubiquitination. *J Biol Chem* **290**, 28760-28777
3. Huang, F., Chandrasekharan, M. B., Chen, Y. C., Bhaskara, S., Hiebert, S. W., and Sun, Z. W. (2010) The JmjN domain of Jhd2 is important for its protein stability, and the plant homeodomain (PHD) finger mediates its chromatin association independent of H3K4 methylation. *J Biol Chem* **285**, 24548-24561

Supplementary Table 2. Primers used in qRT-PCR

Primer	Sequence
PHO5 Sense For	CAGATACGTCATTAACGATGCTGTT
PHO5 Sense Rev	CCGGCTACTCTCTTTTCAGCA
PHO5 Antisense For	CCTCGACTTAGCAAAACATCA
PHO5 Antisense Rev	CGCACATGCCAAATTATCAA
PHO89 Sense For	TCAATTGAACTAGCTGTTGCTATTACT
PHO89 Sense Rev	AACCAACAGCGACAATACCA
PHO89 Antisense For	GTCACTCATTCTATGACAATTCATGT
PHO89 Antisense Rev	TAAGCCCGGTTTTCGATATGA
5'-SER3-For	GACCAAGAGAACACTGTGCGTGTG
3'-SER3-Rev	GAGATCTTAGCAGAGGTTTGATTTAG
5'-CHA1-For	TTGTAATAGAGGACAAAGATGTTATTG
3'-CHA1-Rev	AAATTGTCAGCGACTTCTATTACAGG
LCP5-For	CAGAGAAGGAACGTAGAGTCACTTC
LCP5-Rev	TACAGTCTCCTTTGAGCTCTATCCC
DBP8-For	CATAGATCTGGTCGTACGGCCC
DBP8-Rev	TCAAGAGCGCAAACTTTTCCCGTC
